# Polysulfone Membranes Doped with Human Neutrophil Elastase Inhibitors: Assessment of Bioactivity and Biocompatibility

**DOI:** 10.3390/membranes13010089

**Published:** 2023-01-10

**Authors:** Susana Rocha, Rita Félix, Maria João Valente, Andreia Bento-Silva, Rute Rebelo, Célia Gomes Amorim, Alberto da Nova Araújo, Rui Moreira, Alice Santos-Silva, Maria Conceição B. S. M. Montenegro

**Affiliations:** 1 UCIBIO—Applied Molecular Biosciences Unit, Biochemistry Laboratory, Department of Biological Sciences, Faculty of Pharmacy, University of Porto, 4050-313 Porto, Portugal; 2 Associate Laboratory i4HB—Institute for Health and Bioeconomy, Faculty of Pharmacy, University of Porto, 4050-313 Porto, Portugal; 3 Faculty of Pharmacy, University of Lisbon and Research Institute for Medicines (iMed.ULisboa), 1649-003 Lisbon, Portugal; 4 National Food Institute, Technical University of Denmark, 2800 Kongens Lyngby, Denmark; 5 Faculty of Pharmacy, University of Lisbon, 1649-003 Lisbon, Portugal; 6 LAQV/REQUIMTE, Applied Chemistry Laboratory, Department of Chemical Sciences, Faculty of Pharmacy, University of Porto, 4050-313 Porto, Portugal

**Keywords:** hemodialysis, chronic kidney disease, biocompatibility, 4-oxo-β-lactam compounds

## Abstract

The use of polysulfone (PSU) hemodialysis (HD) membranes modified with bioactive compounds has gained relevance in chronic kidney disease (CKD) management. Compounds based on the 4-oxo-β-lactam scaffold have outstanding inhibitory ability and selectivity for human neutrophil elastase (HNE). The present work aimed to evaluate the bioactivity and biocompatibility of PSU-based HD membranes doped with HNE inhibitors (HNEIs). For this, two 4-oxo-β-lactam derivates (D4L-1 and D4L-2) synthesized in house were used, as well as a commercial HNEI (Sivelestat), for comparison purposes. Their HNE inhibition efficacy was evaluated in in vitro and ex vivo (incubations with human plasma) assay conditions. All biomaterials were bioactive and hemocompatible. The inhibitory capacity of the HNEIs and HNEI-PSU membranes in vitro was D4L-1 > D4L-2 > Sivelestat and D4L-2 > Sivelestat > D4L-1, respectively. In ex vivo conditions, both HNEIs and HNEI-PSU materials presented the same relative inhibitory ability (D4L-1 > D4L-2 > Sivelestat). The difference observed between in vitro and ex vivo conditions is most likely due to the inherent lipophilicity/hydrophobicity of each HNEI influencing their affinity and accessibility to HNE when trapped in the membrane. Compared to Sivelestat, both D4L-1 and D4L-2 (and the respective doped membranes) have more potent inhibition capabilities. In conclusion, this work reports the successful development of PSU membranes functionalized with HNEIs.

## 1. Introduction

Chronic kidney disease (CKD) is associated with a progressive and irreversible loss of kidney function. Patients at the more severe stage of CKD, end-stage renal disease (ESRD) patients, require hemodialysis (HD) treatment or renal transplantation to survive [1,2]. Dialysis treatment carries an increased risk of cardiovascular (CV) morbidity and mortality, driven by traditional risk factors (e.g., age, dyslipidemia, hypertension, and diabetes mellitus) and disease-specific factors (e.g., uremic toxins, vascular calcification, malnutrition, inflammation, and oxidative stress) [2,3,4].

Chronic inflammation, a common feature in CKD, is closely related to its progression and outcome and is particularly enhanced in ESRD patients under HD. Despite the technical advances in the preparation of biocompatible dialysis membranes, this procedure is still accompanied by an inflammatory response [5,6]. The recurrent interaction of blood with the dialysis membranes appears to trigger neutrophil activation, with the production of reactive oxygen species and cytokines, and with the release of its granule contents, rich in cationic proteins and proteases, such as elastase [7,8].

Human neutrophil elastase (HNE, EC 3.4.21.37) is a member of the chymotrypsin superfamily of serine proteases; its proteolytic activity is regulated by potent anti-proteases, namely alpha1-antitrypsin, alpha2-macroglobulin, secretory leukocyte protease inhibitor, serpin B1, and elafin [9]. An imbalance between HNE and its inhibitors results in tissue damage that may lead to organ dysfunction, since HNE has a relevant role in the degradation of extracellular matrix proteins and mediates regulatory functions of the inflammatory response. Thus, elastase may contribute to perpetuating the inflammatory state in patients with CKD and favor the development of other inflammatory conditions. [4,7,8,9,10,11,12]. High levels of elastase have been associated with CV diseases [4,7,8,9,10] and with chronic obstructive pulmonary disease, acute lung injury, cystic fibrosis, and ulcerative colitis [8,9,13].

While the use of HNE inhibitors (HNEIs) as therapeutic tools in CKD is an unexplored avenue, they have been studied for several decades due to their potential applicability in the treatment of cardiopulmonary diseases and other inflammatory pathologies [8,9,13,14,15]. HNEIs range from the 1st generation of natural peptide inhibitors (e.g., alpha 1-proteinase inhibitor, elafin, and secretory leukocyte protease inhibitor); to the 2nd generation of exogenous and synthetic specific inhibitors, such as Sivelestat (ONO-5046; Elaspol^®^ 100, a clinically approved marketed drug); and, more recently, to the 3rd/4th (AZD9668, Alvelestat) and 5th generations (BAY-858501), used in Phase II clinical trials [8,9,13,15,16]. Some reports have suggested a beneficial effect of these molecules on kidney injury in rats [17,18,19,20], and Grano et al. [21] reported the use of membranes coated with the inhibitors alpha1-antitrypsin, serpin B1, elafin, or Alvelestat to reduce the proteolytic activity of HNE in in vitro tests. These studies showed the potential of using immobilized HNEIs on HD membranes as an innovative technological approach for dialysis treatment in CKD patients.

Low-molecular-weight synthetic HNEIs have higher enzyme selectivity, stability, and biocompatibility than the 1st-generation inhibitors, but their most interesting feature is that their molecules’ structure can be easily tweaked to produce new compounds with desirable physicochemical and pharmacokinetic properties [13,14,15,16]. Among these, our team has shown that kojic acid and 4-oxo-β-lactam scaffolds are excellent starting points to design derivates that have outstanding inhibitory ability and selectivity for HNE [16,22,23,24]. As with Sivelestat (ONO-5046), these derivates are all acylating agents with the typical mechanism based on a covalent reversible or irreversible binding, which largely depends on the balance between the acylating power of the inhibitor and the rate of “enzyme deacylation” to regenerate the free enzyme. In particular, 4-oxo-β-lactams have shown to be potent acylating agents, consistent with a strained and reactive 4-member ring [15,16,22].

The two main materials used in dialyzer membranes are the synthetic polymers, polyethersulfone (PES) and polysulfone (PSU) [25,26,27,28], independently of the type of dialysis. In fact, the use of PSU membranes modified with bioactive compounds has gained relevance in clinical settings and CKD management. Beyond their efficiency in the removal of uremic toxins and extra water, and in retaining essential metabolites, these PSU membranes have a specific biological activity to inhibit a dialysis-associated complication. Vitamin E-coated PSU membranes with improved biocompatibility have been introduced in clinical practice [29], followed by several commercial models of cellulose/PSU-based low/high flux coated membranes. In a previous work [30], to improve the effectiveness of PSU-based membranes, different types and concentrations of hydrophilic additives were tested, namely, polyethylene glycol (of different molecular weights) and polyvinylpyrrolidone (PVP). All additives were successfully incorporated [30], but the incorporation of 2.5 wt% of PVP yielded a material with higher hydrophilicity and porosity while maintaining the desirable permeability for low-molecular-weight proteins, mechanical strength, and hemocompatibility. Moreover, it was shown that these membranes, when doped with the antioxidant compounds lipoic acid, vitamin E, or a combination of both, still showed high performance [31].

Given the crucial role of neutrophil activation associated with the HD procedure, the present work aimed to incorporate HNEIs into PSU-based membranes and evaluate their bioactivity and biocompatibility, as well as their efficacy in HNE inhibition in in vitro and ex vivo settings. For this purpose, 4-oxo-β-lactam derivates (D4L-1 and D4L-2) from our internal library and the commercially acquired Sivelestat (as reference) were tested.

## 2. Materials and Methods

### 2.1. Materials

Deionized water Milli-Q (conductivity < 0.1 μS cm^−1^) (Millipore system, Molsheim, France) and analytical grade chemicals were used without further purification unless otherwise stated. PSU in pellets with average molecular weight (MW) 35,000 Da (CAS Number 25135-51-7); 1-methyl-2-pyrrolidone (NMP) (CAS Number 872-50-4); Polyvinylpyrrolidone (PVP) K30 (CAS Number 9003-39-8); HEPES sodium salt (CAS Number 75277-39-3); phorbol-12-myristate-13-acetate (PMA) (CAS Number 16561-29-8); acetonitrile (CAS Number 75-05-8); Elastase, Human Neutrophil (CAS 9004-06-2); Elastase Substrate V, Fluorogenic (CAS 72252-90-5); and single-side polished N-type silicon wafer (CAS Number 7440-21-3) were all purchased from Sigma-Aldrich Co., Saint Louis, MO, USA. Sivelestat, neutrophil elastase inhibitor (CAS Number 127373-66-4), and Human PMN Elastase ELISA Kit were acquired from Abcam, Cambridge, UK. Dimethyl sulfoxide (DMSO) (CAS Number 127373-66-4) was purchased from VWR Chemicals, Radnor, PA, USA. Monoclonal antibodies CD42a (GRP-P), PE, and CD62P (P-Selectin), and APC, used for the determination of platelet activation, were purchased from Invitrogen, ThermoFisher Scientific, Waltham, MA, USA.

### 2.2. Methods

#### 2.2.1. Synthesis of the HNEIs

D4L-1 was prepared as previously described in the literature [32]: 1H NMR (300 MHz, CDCl3) δ 8.38 (d, J = 1.7Hz, 1H), 8.08 (dd, J = 9.0, 1.7Hz, 1H), 7.82 (d, J = 8.5Hz, 2H), 7.54 (d, J = 8.5Hz, 2H), 7.50 (d, J = 9.0Hz, 1 H), 4.57 (s, 2 H), 1.83 (q, J = 7.5 Hz, 4 H), 1.04 (t, J = 7.5 Hz, 6 H); 13 C NMR (75 MHz, CDCl3) δ 172.4, 172.0, 166.4, 155.6, 142.3, 134.7, 130.4, 128.8, 127.1, 126.5, 121.4, 119.8, 110.2, 72.6, 36.4, 24.3, 9.5. ESI-MS (+) *m/z*: 425.0 [M+H]+; Anal. Calcd. (C22H20N2O5S): C, 62.3; H, 4.8; N, 6.6; S, 7.6; O, 18.9%. Found: C, 62.2; H, 4.8; N, 5.9; S, 7.7; O, 19.4%.

D4L-2 was also prepared following the same method [32]: 1H NMR (300 MHz, CDCl3) δ 8.18 (d, J = 9.0Hz, 2H), 7.99 (d, J = 9.0Hz, 2H), 1.89 (q, J = 7.7Hz, 4H), 1.08 (t, J = 7.7Hz, 6H); 13C NMR (75 MHz, CDCl3) δ 172.1, 170.4, 138.3, 131.8, 127.1, 118.9, 72.8, 24.1, 9.4. ESI-MS (+) *m/z*: 260.1 [M+H]+; Anal. Calcd. (C14H15NO4): C, 64.4; H, 5.8; N, 5.4; O, 24.5%. Found: C, 63.8; H, 5.7; N, 5.3; O, 25.2%.

Sivelestat (ONO-5046), a known 2nd-generation clinically approved HNEI, was used as a reference compound in all subsequent assays. It was obtained from its supplier and kept at room temperature under a dry atmosphere.

Stock solutions (1.0 mg mL^−1^) of all HNEIs were prepared in DMSO, aliquoted, and stored at −20 °C until assayed.

#### 2.2.2. Determination of HNEIs IC50

To assess the capacity and selectivity of each compound to inhibit HNE (i.e., bioactivity), a fluorometric assay was carried out, using the fluorogenic substrate MeO-Suc-Ala-Ala-Pro-Val-AMC (Elastase Substrate V), as previously described [23]. For that, HNE was incubated without and with HNEIs for 30 min at 25 °C; afterwards, the substrate was added, and the fluorescence was monitored over another 30 min at 25 °C. In all assays, a saturated substrate concentration was used to ensure a fluorescence signal within the analytical range. The appropriate controls were run (HNE alone, substrate alone, and HNEIs vehicle (2.5% DMSO in HEPES 0.1M buffer, pH 7.4)). At least 5 independent assays (in triplicate) were performed for each HNEI, and the range of HNEIs’ concentrations used covered from none to maximum HNE inhibition (0.1–160.0 nM, 0.1–600.0 nM, and 1.0–900.0 nM for D4L-1, D4L-2, and Sivelestat, respectively); for every assay, freshly prepared HNEIs from the stock solutions were used. HNE activity of HNEI vehicle samples (2.97% ± 0.07 of control) showed no statistically significant difference (*p* = 0.801, Student’s *t*-test) in comparison to the positive control (HEPES buffer, HNE, and substrate), showing that the vehicle by itself had no relevance on HNE activity. Thus, in all assays, the HNE activity value corresponding to the 2.5% DMSO samples was considered to be 100% (maximum) and used to standardize the values obtained for all samples. The IC50 of each HNEI was estimated by applying the logistic regression model for a normalized response, with a 95% confidence interval, utilizing the GraphPad Prism 8 version 8.0.2 for Windows (GraphPad Software, San Diego, CA, USA).

#### 2.2.3. Hemocompatibility of HNEIs

The safety of HNEIs by themselves had to be tested for their potential use on dialysis membranes in HD procedures. The biocompatibility assays described herein were performed as recommended by the International Standard ISO 10993-4 (2017). Whole blood was collected from healthy adult volunteers by venipuncture into tubes containing sodium citrate, as an anticoagulant. All individuals gave informed consent; this study was approved by the ethical committee of FFUP (Reference Nº 37-06-2019) and was performed in compliance with the Declaration of Helsinki.

In all assays, the hemoglobin (Hb) concentration of each blood sample was adjusted to 10 g dL^−1^ with sterile phosphate-buffered saline (PBS), pH 7.4, to standardize the experiments. Two experimental duplicates were performed in each independent assay for all HNEIs (at least three independent assays were executed). Whole blood was incubated alone (blank), with HNEIs’ vehicle (0.125% DMSO), and with each HNEI in the concentrations corresponding to 0.1× IC50, 1× IC50, 10× IC50, and 100× IC50 for 3 h at 37 °C, in 12-well plates (800.0 µL/well). Afterwards, erythrocyte morphology, plasma Hb concentration (hemolysis), and platelet (PLT) activation were assessed as described elsewhere [31]. Briefly, after incubations, Wright-stained [33] blood smears were prepared to study the blood cell morphology by optical microscopy (Nikon Eclipse Ci), and PLT rich plasma and PLT poor plasma samples were obtained by centrifugation (250× *g*, 15 min at 4 °C and 1500× *g*, 5 min at 4 °C, respectively) to evaluate hemolysis and PLT activation, respectively. A spectrophotometric method (540 nm) was used to measure plasma Hb levels utilizing an Hb concentration standard curve (1 to 200 mg dL^−1^). To quantify PLT activation, PLT suspensions were double-stained with monoclonal antibodies for CD42a (GRP-P) conjugated with phycoerythrin (PE), and for CD62P (P-Selectin)-conjugated allophycocyanin (APC), ensuring the simultaneous staining of total PLT and of the activated platelets, respectively. PLT-specific events were analyzed by flow cytometry (BD Accuri C6, BD Biosciences), and PLT activation was determined as the percentage of activated platelets (CD62P-positive events) within the total PLT count (CD42a-positive events).

Vehicle and blank (blood only) samples presented equal values of plasma Hb concentration (vehicle was 0.014% ± 0.002 of blank; *p* = 1.000, Student’s *t*-test) and PLT activation (vehicle was −0.706% ± 0.004 of blank; *p* = 0.936, Mann–Whitney U test), confirming that the HNEI vehicle was hemocompatible. Thus, the plasma Hb levels and PLT activation (%) of HNEIs samples were normalized by subtracting the respective values from the vehicle samples (to correct the effects caused by the incubation process per se).

#### 2.2.4. Bioactivity Kinetic Assay of HNEIs on Human Plasma

The bioactivity of the HNEIs on human plasma (ex vivo conditions) was also tested. The same concentrations of HNEIs used in the hemocompatibility assays were used to evaluate their efficacy in plasma, over different incubation times, at 37 °C. To carry out these assays, a pool of human plasma was obtained from venous blood (EDTA as anticoagulant and centrifuged at 2000× *g*, 4 °C, 15 min); this plasma pool was a mix obtained from blood samples from healthy individuals and ESRD patients on HD.

Plasma and plasma diluted with PBS, pH 7.4 (50%), were used to evaluate medium hydrophobicity effects; the elastase concentration of both plasma and diluted plasma was 41.44 ± 4.16 ng mL^−1^ and 17.02 ± 2.01 ng mL^−1^, respectively (measured by enzyme-linked immunosorbent assay for human PMN elastase).

At least three independent assays (with experimental triplicates) were performed for each HNEI. Plasma or diluted plasma were incubated alone (blank), with HNEIs’ vehicle (0.125% DMSO), and with each HNEI in the concentrations corresponding to 0.1×, 1×, 10×, and 100× IC50, at 37 °C for different time points (0, 15, 30, 60, and 180 min), in 96-well plates (200.0 µL/well). In parallel, the vehicle and the studied HNEIs at 1× and 100× IC50 concentrations were incubated in HEPES 0.1M buffer, pH 7.4, as a reference from the in vitro assays. After each time point, the HNE activity was measured by fluorimetry as described above [23], with slight adjustments to the total volume in the well (2.5× less than the original technique), in order to provide reliable results when applied in plasma. Performing this assay at the end of each time point ensured that the exogenous recombinant HNE added reacted with the HNEIs that were present and available in the solution after the different time incubations, thus assessing the activity of HNEIs in each medium (plasma, diluted plasma, or HEPES buffer) over time.

The HNE activity of vehicle samples in plasma (4.66% ± 0.03 of blank), diluted plasma (−1.44% ± 0.03 of blank), and HEPES buffer 0.1M, pH 7.4 (−6.90% ± 0.05 of blank), showed no statistically significant differences in comparison to blank samples (*p* = 0.570, *p* = 0.881, *p* = 0.586, respectively; Student’s *t*-test). Therefore, in all assays, the HNE activity value corresponding to vehicle samples was considered to be the maximum, equivalent to no HNE inhibition (0%). This was used to calculate and standardize the percentage of HNE inhibition for the HNEIs samples.

#### 2.2.5. Preparation of HNEI-PSU Membranes

PSU membranes were prepared through the spin-coating technique, followed by the phase inversion coagulation method according to Kohlová et al. [30]. The optimized composition of the flat-sheet membranes was 15.0 wt% of PSU (main polymer), 2.5 wt% of PVP (hydrophilic additive), and 82.5 wt% of NMP (solvent); they were stored at 4 °C, protected from light and humidity (silica gel desiccant packets), until enrichment with the inhibitors.

The immobilization of Sivelestat, D4L-1, and D4L-2 was achieved through the adsorption method: PSU membranes were cut into circles of 2.0 cm in diameter (VLS laser platform; LaserMaq, Portugal) with an average weight of 2.77 ± 0.25 mg, placed in 12-well plates (triplicates), and incubated at 25 °C for 3 h with 1.0 mL of ultrapure water (blank), with 2.5% DMSO solution (vehicle), and with 5, 25, 50, 250, 500, and 1000 nM solutions of each HNEI. The HNEI solutions were prepared immediately before the assay from stock solutions. After incubation, the membranes were dried (tissue paper and air) to remove the excess of solution contained in the pores. All ensuing experiments with the HNEIs-PSU membranes described herein were carried-out within 24 h after the adsorption process.

#### 2.2.6. Characterization of HNEI-PSU Membranes

The HNEIs’ immobilization effectiveness was assessed by determining the HNE inhibitory capacity (i.e., bioactivity) of the HNEIs-PSU membranes, and by quantifying the amount of HNEI bound to the membrane (adsorption yield), by ultra-high-performance liquid chromatography coupled with tandem mass spectrometry (UHPLC-MS/MS), as reported below.

##### Bioactivity (In Vitro)

The elastase inhibition ability of the blank-PSU, vehicle-PSU, and HNEIs-PSU membranes prepared as described above (Section 5) was determined in three independent assays (in triplicates). The HNE activity assay of the modified materials was performed as previously described (Section 2) [23], with a slight adaptation: instead of solutions, circles (Ø = 0.6 cm, cut with a hole punch) of the membranes were placed into the wells of the 96-well plates.

The bioactivity of PSU membranes incubated with the vehicle (2.5% DMSO) showed no statistically significant difference in comparison to the blank samples (−4.33% ± 0.10 of blank; *p* = 0.767, Student’s *t*-test). For normalization of the results, the percentage of HNE inhibition was calculated and standardized considering that the PSU membranes in contact with the vehicle (2.5% DMSO) corresponded to no HNE inhibition (0%).

##### Adsorption Extension

The extension of HNEIs’ adsorption (in %) to the PSU membranes was determined by evaluating the difference of the amount of compound in the solution in contact with the membrane before and after incubation. Aliquots (800.0 µL) of the initial and final HNEI and vehicle (control samples) solutions were transferred to Eppendorf tubes and frozen at −80 °C for further lyophilization (LyoQuest; Telstar, Terrassa, Spain). The amount of each HNEI was assessed by UHPLC-MS/MS, dissolving the tubes’ lyophilized content in 100 µL of acetonitrile:water (75:25). The solutions were homogenized in the vortex, sonicated for 10 min, filtered through 0.20 µm PTFE syringe filters (Labfil, Shaoxing, China), and injected into the equipment. Samples were prepared in triplicate.

A kinetics study was performed through Freundlich adsorption isotherm to determine the adsorption capacity (K_F_) and intensity of adsorption (n). This isothermal empirical model was chosen because, according to Kohlová et al. [30], the material presented an uneven pore size between the two surface layers and was composed of a blend of PSU and PVP, thus presenting many and different types of available surface adsorption sites, each one with individual free energy of adsorption on the liquid–solid interface [34].

##### UHPLC-MS/MS Conditions

The UHPLC analyses were performed on an Acquity^TM^ Ultra Performance LC (Waters, Dublin, Ireland) equipped with a binary pump, solvent degasser, auto sampler, and column oven. The separation was performed on a reversed-phase column (Purospher^®^ STAR RP-18 2 μm, 2.1 × 50 mm; Merck, Darmstadt, Germany) at 35 °C, using an injection volume of 10 μL. The mobile phase consisted of Milli-Q water containing 0.1% formic acid (A): acetonitrile (B) at a flow rate of 0.30 mL min^−1^. The following gradient of eluents was used: 30% B for 1 min, from 30 to 95% B for 5 min, held isocratically at 95% B for 4 min, and finally returning to the initial conditions for 5 min. Tandem mass spectrometry (MS/MS) detection was performed on an Acquity^TM^ triple quadrupole (Waters, Dublin, Ireland) using an electrospray ionization (ESI) source operating in negative ion mode at 120 °C and applying a capillary voltage of 3.0 kV. High-purity nitrogen (N_2_) was used both as a drying gas and as a nebulizing gas, and ultra-high purity argon (Ar) was used as a collision gas. MS/MS conditions were optimized for D4L-1, D4L-2, and Sivelestat, which were analyzed in multiple reaction monitoring (MRM) mode, to achieve a higher selectivity and sensitivity. Two transitions were used in order to quantitate (MRM1) and confirm the identification (MRM2) of each compound (Table S1 of Supplementary Materials), with a maximum deviation of 15% between the MRM1/MRM2 ratio. MassLynx software, version 4.1 (Waters, Dublin, Ireland) was used to control the system, for data acquisition and processing.

Calibration curves of all compounds were prepared in three blank matrixes (control samples prepared as previously described), from 1 to 30 ng mL^−1^ for D4L-2 and Sivelestat, and from 2 to 35 ng mL^−1^ for D4L-1. The best fit for the calibration curves presented correlation coefficients (R^2^) higher than 0.99. To determine the limit of detection (LOD) of each compound, five independent stock solutions were diluted in a blank matrix, until a signal-to-noise ratio of 3:1. The LOD obtained for Sivelestat was 0.1 ng mL^−1^ (corresponding to 0.01 ng in the lyophilized sample), and for both D4L-1 and D4L-2 it was 0.2 ng mL^−1^ (0.02 ng). Similarly, five independent stock solutions were diluted in a blank matrix to determine the limit of quantitation (LOQ) of each compound, which corresponded to the lowest concentration to give acceptable (<20%) precision and accuracy errors. The LOQ obtained for both D4L-2 and Sivelestat was 1 ng mL^−1^ (0.1 ng in the lyophilized sample) and for D4L-1 was 2 ng mL^−1^ (0.2 ng). To control the signal of the equipment, standard mixtures of the three compounds at 1 and 10 ng mL^−1^, prepared in a blank matrix, were analyzed after every twenty injections. Precision and accuracy errors were below 10% for all compounds at 10 ng mL^−1^, and below 20% at 1 ng mL^−1^ for D4L-2 and Sivelestat.

#### 2.2.7. Biocompatibility of HNEI-PSU Membranes

Since the final objective of this work is the potential use of these modified membranes in HD procedures, it is mandatory to evaluate their biocompatibility. The hemocompatibility of the bioactive HNEI-PSU membranes was evaluated according to the International Standard ISO-10993-4 (2017), as was aforementioned. Three independent assays (in duplicates) were performed utilizing blank-PSU, vehicle-PSU, and HNEI-PSU membrane circles (Ø = 2 cm) incubated with 1.0 mL of whole blood (Hb 10 g dL^−1^; citrate as anticoagulant) for 3.5 h at 37 °C, in 12-well plates. Afterwards, the RBC morphology, plasma Hb concentration, and platelet activation were determined as described above [31]. Since vehicle- and blank-PSU membranes samples showed similar values of plasma Hb concentration (vehicle was 2.39% ± 0.09 of blank; *p* = 0.812, Student’s *t*-test) and of PLT activation (vehicle was 0.73% ± 0.35 of blank; *p* = 0.988, Mann–Whitney U test), the plasma Hb levels and PLT activation (%) of the HNEI-PSU samples were normalized by subtracting the respective values from the vehicle-PSU samples.

#### 2.2.8. Bioactivity Effectiveness of HNEI-PSU Membranes on Human Plasma

Similar to what was performed with the free HNEIs in human plasma (Section 4), their inhibitory ability was also evaluated when incorporated into PSU membranes. At least three independent assays (in experimental triplicates) were performed for each HNEI. In these sets of experiments, blank-, vehicle-, and HNEIs-PSU membrane circles (Ø = 0.6 cm) were incubated with 200.0 µL of diluted plasma (50% with PBS, pH 7.4) or HEPES buffer 0.1M, pH 7.4, for 3 h at 37 °C. The fluorometric assay to measure HNE activity was performed as described above; the bioactivity of the membranes was measured before and after the incubations, as well as the bioactivity of the plasma and HEPES buffer at the end of the incubations (to evaluate leaching).

The HNE activity of vehicle-PSU membranes incubated in diluted plasma (−0.55% ± 0.16 of blank) or HEPES buffer (2.01% ± 0.05 of blank) showed no statistically significant differences in comparison to blank-PSU membrane samples (*p* = 0.981 and *p* = 0.800, respectively; Student’s *t*-test). The HNE activity at the end of the incubations of the diluted plasma (vehicle 6.58% ± 0.30 of blank; *p* = 0.885, Student’s *t*-test) or the HEPES buffer (vehicle 9.03% ± 0.37 of blank; *p* = 0.866, Student’s *t*-test) in contact with vehicle- or blank-PSU membranes were not statistically different. Thus, the percentage of inhibition of HNE was calculated and standardized, establishing that the inhibition of the membrane when in contact with the vehicle was null (0%).

#### 2.2.9. Statistical Analysis

A statistical analysis was carried out using the Statistical Package for Social Sciences (SPSS, version 28.0, Chicago, IL, USA) for Windows. The Shapiro–Wilk test was used to assess the normality of data distribution. Data were presented as mean ± SEM (standard error of the mean). To compare sets of data that presented Gaussian distribution, the one-way ANOVA test (coupled with Bonferroni post-hoc) or the Student’s unpaired *t*-test were used; to compare sets of data that did not present Gaussian distribution, the Kruskal–Wallis test or the Mann–Whitney U test were chosen. A *p* < 0.05 was considered statistically significant.

## 3. Results

### 3.1. HNEIs

#### 3.1.1. HNEIs’ Characterization

Novel molecules with the oxo-β-lactam motif recently developed as potent and selective HNEIs [32] were selected to perform this study due to their favorable aqueous solubility and lipophilicity. The chemical characteristics of D4L-1, D4L-2, and Sivelestat are presented in Table 1. The latter, which is clinically approved and commercially available, was used as a reference in all experiments [13,14,15,16]. D4L-1 presented the highest CLogP and the lowest LogS values, respectively, whereas, D4L-2 had the lowest molecular weight, tPSA, and cLogP values; pKa values were similar for all compounds (Table 1).

The in vitro bioactivity of each HNEI was assessed through an HNE activity assay that was used to estimate the half-maximal inhibitory concentration, IC50_HNE_ (with a 95% confidence interval), as shown in Figure S1 of Supplementary Materials. IC50 values of D4L-1, D4L-2, and Sivelestat were 10.8 (10.3–11.3) nM, 18.0 (16.5–19.8) nM, and 25.7 (19.1–33.6) nM, respectively, which were significantly different (*p* < 0.001).

Concerning biocompatibility, for all compounds’ concentrations, the induced hemolysis was well below the cut-off value (10.0 mg dL^−1^) for hemolytic behavior [ISO 10993-4 (2017)], and PLT activation was similar to the vehicle samples (Figure S2 of Supplementary Materials). In accordance, the morphology of red blood cells (RBCs) was not altered by any of the HNEIs, as can be observed in Figure S3 of Supplementary Materials.

#### 3.1.2. Ex Vivo Biological Effectiveness of HNEIs in Solution

After verifying the in vitro bioactivity of the HNEIs (in 0.1M HEPES buffer, pH 7.4) and determining their IC50, their inhibitory capacity in human plasma was evaluated. Indeed, using only highly pure recombinant HNE (in the appropriate conditions of pH and temperature) in the in vitro conditions may not reflect what occurs in vivo. This test aimed to ascertain if the behavior of the inhibitors in vitro was similar in human plasma (ex vivo assay). For that, a pool of plasma obtained from healthy individuals and ESRD patients on HD was used, ensuring that the inhibitors would be studied in a medium closer to “real” circumstances ((patho)physiological conditions), namely, in the presence of endogenous serine proteases (neutrophil elastase, proteinase-3, and cathepsin G) and anti-proteases (alpha1-antitrypsin, elafin, secretory leukocyte protease inhibitor, etc.), as well as a myriad of other plasma proteins and components [8,9,14]. To evaluate the effect of the assay’s medium (environment), these experiments were performed with undiluted and with diluted plasma (50%; 0.1M HEPES buffer, pH 7.4). Moreover, to standardize the values of bioactivity, it was established that the values from vehicle samples corresponded to 0% inhibition, thus “eliminating/leveling” any plasma’s endogenous/native HNEIs’ contribution that could affect the activity of the added (exogenous) HNE.

**Figure 1:**
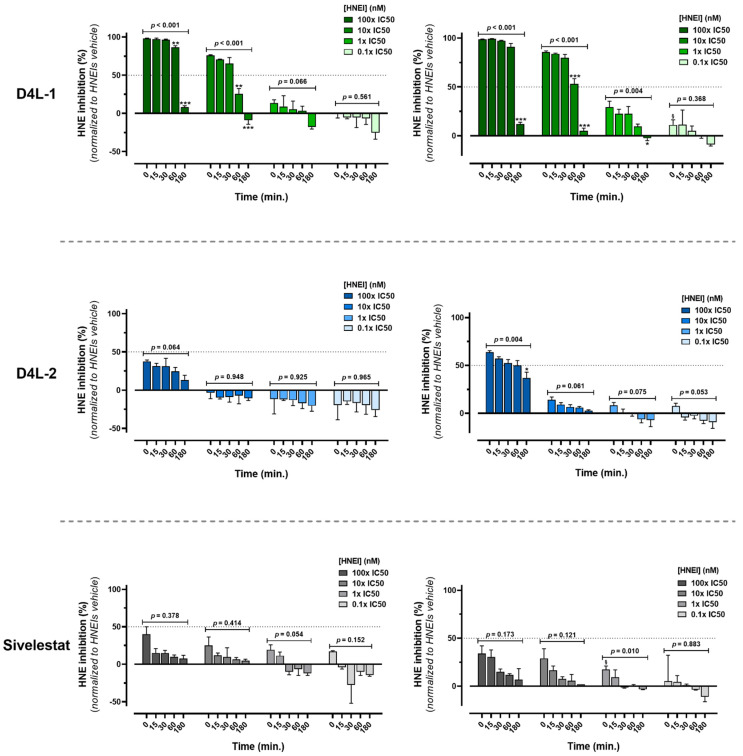
Human neutrophil elastase (HNE) inhibition of D4L-1, D4L-2, and Sivelestat when incubated with plasma (left side) or diluted plasma (right side), assessed by the HNE activity assay after incubations of 0, 15, 30, 60, and 180 min at 37 °C. Data obtained from at least 3 independent experiments (performed in triplicate) are presented as mean ± SEM (standard error of the mean). Dotted line represents the IC50 value of the respective HNEI. Brackets denote the statistical analysis results of time of incubation comparison for the respective solution concentration of each HNEI (one-way ANOVA test), * *p* < 0.05, ** *p* < 0.01, *** *p* < 0.001 between that time point and all the preceding time points, ^§^ *p* < 0.05 between 0 min and 60/180 min (Bonferroni post hoc test); *p* < 0.05 was considered statistically significant. For each assay, HNE inhibition was standardized establishing that HNEIs’ vehicle (0.125% DMSO) samples corresponded to zero (minimum). HNEI, human neutrophil elastase inhibitor; IC50, half maximal inhibitory concentration.

The inhibitory capacity of all HNEIs differed greatly from the in vitro results (IC50, Figure S1 of Supplementary Materials), since in plasma (diluted or not) none of them reached 50% of inhibition at 0 min for the 1 × IC50 concentration (represented by dotted lines) (Figure 1 and Table S2 of Supplementary Materials). The inhibitory capacity of all HNEIs decreased in both plasma and diluted plasma along the incubation time, with this decrease being less prominent for the latter medium. In any given concentration, the best performing HNEI was D4L-1, showing consistently the highest HNE inhibition (plasma and diluted plasma), followed by D4L-2, and finally Sivelestat. Comparing plasma and diluted plasma (medium effect), HNE inhibition was greater in diluted plasma for any given HNEI and/or concentration.

When incubated in plasma, D4L-1 at 0.1× IC50 showed no inhibitory capacity at any time point, which was also null for 10× IC50 and 1× IC50 at 180 min; when incubated in diluted plasma, no HNE inhibition was observed for 1× IC50 at 180 min and after 60 min for 0.1× IC50. In plasma, bioactivity was observed for D4L-2 only at the 100× IC50 concentration (at any time point); in diluted plasma, D4L-2 showed no inhibitory ability after 30 min and after 15 min for 1× IC50 and 0.1× IC50, respectively. Sivelestat, in both plasma and diluted plasma, showed no HNE inhibition after 30 min for 1× IC50; for the 0.1× IC50 concentration, this effect was observed after 15 min and after 60 min in plasma and diluted plasma, respectively (Figure 1 and Table S2 of Supplementary Materials).

When incubated in the HEPES buffer, the inhibitory capacity of all HNEIs was similar for all time points and corresponded to 100% and 50% in the 100× and 1× IC50 concentration, respectively (Figure S4 of Supplementary Materials). This control experiment shows that the inhibitors do not lose efficacy over time when the assay is performed in HEPES.

### 3.2. HNEI-PSU Membranes

#### 3.2.1. HNEI-PSU Membranes’ Characterization

Flat-sheet PSU membranes were prepared as described above [30], and the immobilization of the HNEIs by adsorption was studied after their immersion in inhibitor solutions at concentrations ranging from 5 to 1000 nM; this range was chosen to cover the values between 0.1× and 100× IC50 of all molecules (Figure S1 of Supplementary Materials). The efficacy and extension of adsorption were evaluated by measuring the bioactivity of the HNEIs-PSU membranes and by the determination of the amount of the adsorbed inhibitor.

**Figure 2:**
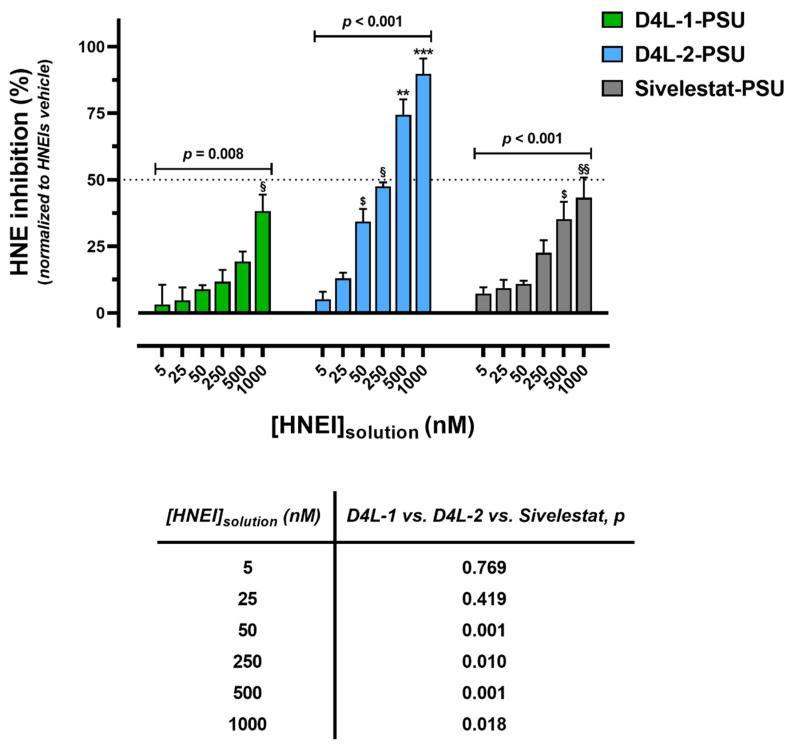
Human neutrophil elastase (HNE) inhibition of D4L-1-PSU, D4L-2-PSU, and Sivelestat-PSU doped membranes by adsorption (25 °C, 3 h), assessed by the HNE activity assay. Data obtained from 3 independent experiments (performed in triplicate) are presented as mean ± SEM (standard error of the mean). Dotted line represents the IC50 value of the respective HNEI in HEPES 0.1M, pH 7.4 buffer. Brackets denote the statistical analysis results of comparison of HNEI-PSU membranes incubated with the respective initial solution concentration of each HNEI (one-way ANOVA test), ** *p* < 0.01, *** *p* < 0.001 between that HNEI solution and all the preceding concentrations; ^§^ *p* < 0.05, ^§§^ *p* < 0.01 between 1000 nM and 5/25 nM; ^$^ *p* < 0.05, between 50 nM and 5 nM. (Bonferroni post hoc test) and the embedded table shows the comparison between D4L-1, D4L-2, and Sivelestat groups within each concentration (one-way ANOVA test); *p* < 0.05 was considered statistically significant. For each assay, HNE inhibition was standardized establishing that PSU membranes incubated with HNEIs’ vehicle (2.5% DMSO) samples corresponded to zero (minimum). HNEI, human neutrophil elastase inhibitor; PSU, polysulfone.

Figure 2 shows the results of the inhibitory ability of the HNEI-PSU membranes, and, as can be seen, all the produced biomaterials were bioactive and the inhibition capacity increased with the increase in the inhibitor concentration of the solutions used for the adsorption process. D4L-1 presented the lowest values of HNE inhibition for any given concentration, while D4L-2 showed the highest. Only the 500–1000 nM D4L-2-PSU membranes showed an HNE inhibitory capacity higher than 50%.

A UHPLC-MS/MS method was successfully developed for the quantitation of DL-1, DL-2, and Sivelestat. Aliquots of the HNEI solutions before and after contact with the PSU membranes were analyzed. A graphic representation of the Freundlich isotherm was plotted, through which the adsorption capacity and extent of adsorption of the bioactive materials under study were determined (Table 2 and Figure S5 from Supplementary Materials). Independently of the concentrations tested, the adsorbed percentage of D4L-1 (roughly 99%) and of Sivelestat (approximately 90%) to the PSU membrane was constant, while the D4L-2 adsorption yield decreased from 98.2% to 86.5%, as the concentration of HNEIs in the solution increased. D4L-1-PSU membranes showed the greatest adsorption capacity (K_F_) and D4L-2-PSU the lowest; the lowest intensity of adsorption (n) was observed for D4L-1-PSU membranes and the highest for D4L-2-PSU (Table 2).

Material-induced hemolysis, PLT activation, and cell morphology were evaluated to assess the hemocompatibility of the developed membranes, which is paramount as they should have the potential to be used as HD membranes. Each HNEI-PSU membrane was tested for concentrations ranging from 5 to 1000 nM of the inhibitor’s initial solution concentration. For all biomaterials, the hemolysis was below the cut-off value considered hemolytic (10.0 mg dL^−1^), and PLT activation was similar to the vehicle (Figure S6 from Supplementary Materials). The morphology of RBCs was not altered by any of the HNEI-PSU membranes, in comparison to the vehicle and blank biomaterials (Figure S7 of Supplementary Materials).

#### 3.2.2. Bioactivity Effectiveness of HNEI-PSU Membranes

The inhibitory capacity of the different membranes doped with HNEIs was studied when they were exposed to plasma (or to HEPES buffer). Considering the results obtained for HNEIs in solution, only two time points were selected, 0 and 180 min, as these times showed the greatest changes in HNE activity; likewise, plasma diluted with PBS was chosen, since HNE inhibition was more readably assessed in this medium (Figure 1). In addition, the assessment of leaching of HNEIs from membranes was assessed by measuring HNE activity from the diluted plasma and HEPES buffer supernatants at the end of the incubations.

**Figure 3:**
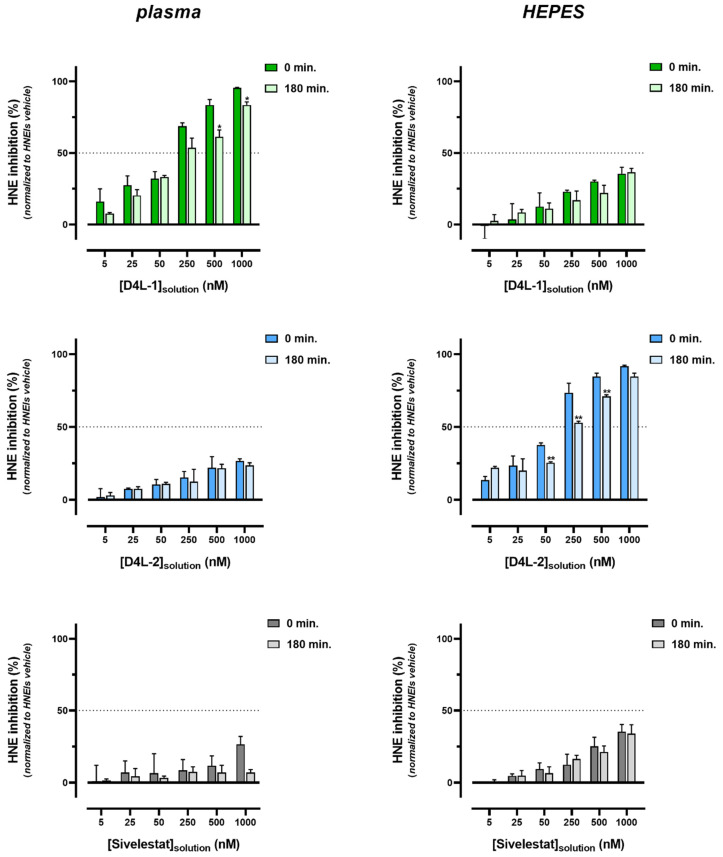
Human neutrophil elastase (HNE) inhibition of D4L-1-PSU, D4L-2-PSU, and Sivelestat-PSU membranes after incubations of 0 and 180 min with diluted plasma (left side) or HEPES buffer (right side) at 37 °C, assessed by the HNE activity assay. Data obtained from at least 3 independent experiments (performed in triplicate) are presented as mean ± SEM (standard error of the mean). Dotted line represents the IC50 value of the respective HNEI in HEPES 0.1M, pH 7.4 buffer. * *p* < 0.05, ** *p* < 0.01 statistical analysis results of time of incubation comparison for the respective initial solution concentration of each HNEI-PSU doped membrane (Student’s *t* test); *p* < 0.05 was considered statistically significant. For each assay, HNE inhibition was standardized establishing that PSU membranes incubated with HNEIs’ vehicle (2.5% DMSO) samples corresponded to zero (minimum).

After 3 h incubation, all biomaterials showed bioactivity, although a shift (in some cases significant) to decreased values was observed for both incubations in plasma and HEPES buffer. The best performing HNEI-PSU material in diluted plasma was D4L-1-PSU, and in the HEPES buffer it was D4L-2-PSU; Sivelestat-PSU presented the lowest HNE inhibition values in both mediums (Figure 3 and Table S3 of Supplementary Materials).

Leaching was observed for all types of membranes in the HEPES buffer and in diluted plasma, since HNE inhibition was observed in both, except for the plasma incubated with 5 to 250 nM Sivelestat-PSU membranes; leached D4L-1 showed the highest inhibitory capacity in diluted plasma, while D4L-2 had the highest in the HEPES buffer (Figure S8 of Supplementary Materials).

## 4. Discussion

This work aimed to dope flat-sheet PSU membranes with small synthetic molecules, specifically, 4-oxo-β-lactam-based HNEIs, to produce successful bioactive HD biomaterials. As far as we know, this is the first report about the incorporation of this type of HNEIs into PSU membranes. Previous studies [21] referred to the immobilization of different elastase inhibitors using Nylon membranes.

Two 4-oxo-β-lactam-based compounds from our in-house library, D4L-1 and D4L-2, and the commercial Sivelestat were selected as candidate HNEIs to graft onto PSU membranes. Based on a previously optimized membrane composition, consisting of PSU and PVP additive [30] (which increases the hydrophilicity of the material), the two new HNEIs were designed proposedly to have specific chemical characteristics: the D4L-1 compound is more lipophilic, while the D4L-2 is the most hydrophilic; Sivelestat presents an intermediate value concerning its lipophilicity and hydrophobicity (Table 1).

Both 4-oxo-β-lactams are potent HNEIs and have in common an N-aryl moiety, which has been modified with a simple carboxyl group (D4L-2), or a more complex benzoxazole ring thiomethyl group and a carboxyl group (D4L-1), to improve solubility and reduce toxicity. While the ethyl groups at C-3 of the 4-oxo-β-lactam moiety sit in the S1 binding pocket, driving the selectivity towards HNE, the *N*-substituents point to the enzyme surface and do not interact with any of the main binding pockets [22]. The structural modifications performed in the *N*-substituents to improve the physicochemical properties did not significantly affect the inhibitory potency against HNE, as expected.

The HNE inhibitory capacity of each HNEI was determined in vitro to compare their bioactivity. Compared to Sivelestat, both in house inhibitors, D4L-1 and D4L-2, were better inhibitors (lower IC50), and D4L-1 was more potent than D4L-2 (Figure S1 of Supplementary Materials). Previous results showed that 4-oxo-β-lactam derivates containing the N-aryl moiety present irreversible inhibition of HNE [22], while Sivelestat has a proven reversible mechanism [35]. Moreover, the derivates with the benzoxazolethiomethyl group give rise to molecules with a very high HNE inhibitory capacity [22], which is in accordance with the results herein. We must note that Sivelestat (ONO-4056) was originally described as having IC50 of 44 nM [36]; however, the one purchased was labeled by the supplier as having IC50 between 19 and 49 nM. For accuracy in our results, the HNE inhibition of Sivelestat was assayed in parallel with D4L-1 and D4L-2. Further characterization of the inhibitors included the assessment of their safety by performing biocompatibility tests with human blood. All HNEIs were found to be hemocompatible, as they did not induce RBC morphological changes, hemolysis, or PLT activation, even at concentrations as high as 100× the IC50 value of each compound (Figures S2 and S3 of Supplementary Materials).

In the tested in vitro assay conditions, all the compounds behaved as selective and potent HNEIs, but previous works [22,24] have reported that the stability of 4-oxo-β-lactam derivates in aqueous solutions is relatively moderate (the best half-life being 37 h). Moreover, the stability of these HNEIs is even more uncertain in plasma, most likely due to their reactivity with off-target plasma components, such as other serine proteases and esterases [22,24]. Therefore, it was found useful to evaluate the behavior of the compounds in complete or diluted plasma, as this was a simple means of adjusting experimental conditions for different endogenous levels of HNE (and other serine proteases) and for different solubility of the compounds. These ex vivo assay results, shown in Figure 1, indicate that the reactivity of all compounds is time-, HNEI concentration-, and medium-dependent. For most cases, at the lowest concentration used (0.1× IC50) the HNEIs did not show the inhibition of HNE in plasma (undiluted), which suggests that they reacted immediately and completely with endogenous HNE and/or with other enzymes, probably because the amount of HNEI added was overwhelmed by the endogenous plasma components. For D4L-1 and Sivelestat, as the HNEI concentration increased, the HNE inhibition also increased, whereas D4L-2 showed inhibitory activity only at the highest concentration (100× IC50), possibly because more and more inhibitors were still “free” as the concentration increased, that are capable of inhibiting the newly added exogenous HNE. The highest inhibitory activity was presented by D4L-1, showing more than 50% of HNE inhibition at 10× IC50 nM, and almost 100% inhibition at 100× IC50 nM; Sivelestat showed inhibitory activity at all concentrations, although it was always less than 50%. When using diluted plasma, the inhibitory capacity for all HNEIs was always higher when compared to (undiluted) plasma, regardless of incubation time or HNEI concentration, which is likely due to the lower concentration of plasma components, including elastase. This ratio of HNEIs/serine proteases also changed along the incubation period as it was observed that the HNE inhibition ability decreased as the time of incubation increased, as shown by the decreasing ability of HNE inhibition (Figure 1). In opposition, the parallel experiments conducted in the HEPES buffer showed that all HNEIs were relatively stable and maintained their inhibitory capacity (100% and 50%) in the aqueous medium at least during 180 min (Figure S4 of Supplementary Materials).

None of the HNEIs reached 50% of inhibition for the 1× IC50 concentration when incubated in plasma (or diluted plasma) at 0 min of incubation, probably due to their quick and high reactivity in this medium (Figure 1). Comparing the three HNEIs, D4L-2 appeared to be the less stable in biological conditions (probably the most reactive or capable of more off-target interactions), since only at the highest concentration some of it remained free to react with exogenous HNE. In contrast, D4L-1 seemed to be the less reactive (or capable of off-target reactions), resisting longer in plasma, while Sivelestat stood in the middle of the other two. The observed results can be attributed to the chemical properties of the compounds (Table 1), especially if the LogP and LogS values are taken into account, which show that D4L-1 is the most lipophilic of the HNEIs and D4L-2 the most hydrophilic. These results did not exclude any of the studied HNEIs as candidates to be used in the preparation of bioactive PSU membranes, since their immobilization could significantly alter their inhibitory properties.

The newly developed HNEI-PSU membranes were capable of HNE inhibition; in fact, D4L-1-PSU, D4L-2-PSU, and Sivelestat-PSU biomaterials showed bioactivity for the entire range of HNEIs’ concentrations tested (Figure 2). For all compounds, the inhibitory ability of the HNEI-PSU membranes significantly increased with the concentration of the initial HNEI solution. D4L-2-PSU membranes presented the highest bioactivity values, while D4L-1-PSU and Sivelestat-PSU showed lower and similar bioactivity. The inhibitory capacity (IC50) of HNEIs in solution was different from that of the respective HNEI-PSU materials. Thus, the following bioactivity sequence for HNEIs in solution was: D4L-1 > D4L-2 > Sivelestat (Supplementary Materials Figure S1) while, when immobilized in the membranes, it was: D4L-2-PSU > Sivelestat-PSU > D4L-1-PSU (Figure 2). Furthermore, although the membranes were doped with concentrations of HNEIs much higher than the respective IC50s, none of the biomaterials produced was able to cause 50% inhibition of HNE (except for the 500 and 1000 nM D4L-2-PSU membranes). Since the adsorption of HNEIs to the PSU membrane was reasonable (Table 2), the bioactivity of the HNEI-PSU membranes > 25 nM should have been greater, at least in theory. This suggests that the availability of the inhibitors to act on exogenous HNE might be hindered when immobilized in the membranes, or their bioactivity might be altered due to the grafting process itself. In accordance with our data, a decreased inhibition activity of HNEIs when immobilized onto Nylon membranes for α1-antitrypsin, bovine pancreatic trypsin inhibitor (BPTI), or elastatinal was reported by Grano et al. [21].

To better understand the adsorption process, the adsorption yields of the HNEIs on the PSU membranes were calculated, as well as the adsorption capacity (K_F_) and extent of adsorption (Table 2 and Figure S5 of Supplementary Materials). Regardless of the concentration of the HNEI solution used, yields of about 99% were obtained for D4L-1, while for Sivelestat the value was lower (about 90%). This shows that both were able to bind to the membrane, reaching almost its maximum capacity. Comparatively, for D4L-2, a gradual decrease in the adsorbed amount of the inhibitor was observed as the concentration of the solution used to incubate the membranes increased. This suggests that, unlike the other inhibitors, the adsorption capacity of the PSU membranes was lower for this compound (Table 2). In accordance, K_F_ was very high for D4L-1, followed by Sivelestat, and lastly by D4L-2 (Table 2). In contrast, for the intensity or extent of adsorption, the most favorable adsorption process occurred for D4L-2/PSU (n > 1), showing the relative ease in which PSU adsorbed D4L-2, in comparison with the other two HNEIs. The results obtained predict that the available adsorption sites and the specific surface area are different for D4L-1, D4L-2, and Sivelestat, being related to hydrophobic characteristics conferred by the nature of the PSU and by the hydrophilic PVP used in the preparation of the membrane. The combined effect of the intrinsic hydrophilicity and the molecular size of the HNEI compounds may be responsible for the results, as D4L-2 had a much lower molecular weight and higher LogS than D4L-1 and Sivelestat (Table 1). Consequently, the spatial distribution and orientation of each HNEI within the membrane will be distinct when meshed upon the membrane scaffold. The membranes are made up of materials with different hydrophilic/lipophilic characteristics (PSU and PVP) and different amounts of each, and, the inhibitors are likely to have preferred binding sites in accordance with their chemical characteristics. It is, therefore, possible that D4L-2 is preferentially adsorbed to the more superficial layer (hydrophilic), being more available to react with HNE, which makes it the more potent inhibitor when entrapped in the membrane (Figure 2), when in aqueous environments.

As the biocompatibility of dialysis membranes is an essential feature in the HD procedure [25,26,27,28], the hemocompatibility of the HNEI-PSU biomaterials was evaluated. All of them were within the safety values regarding plasma Hb concentration, PLT activation, and cell morphology (Figures S6 and S7 of Supplementary Materials), ensuring that our functionalized membranes have the potential for clinical application.

Since the ultimate goal of this work is to develop HD membranes, performing ex vivo assays through the incubation of these biomaterials in plasma was justified. In the ex vivo tests, the inhibitors, both in solution (Figure 1) and immobilized on membranes (Figure 3), showed to have the same behavior, according to the medium in which the incubations occurred. When incubated in plasma, D4L-1-PSU membranes showed the highest bioactivity, while when incubated in the HEPES buffer, D4L-2-PSU presented the best results; Sivelestat-PSU showed intermediate bioactivity in both environments. This can be justified by the degree of hydrophobicity of each molecule. For all HNEIs, and in both mediums, leaching was observed after 3 h of incubation in plasma or the HEPES buffer (Figure S8 of Supplementary Materials). Considering that the amount of loss (leaching) of surface-adsorbed D4L-2 was statistically significant, in comparison with D4L-1/Sivelestat, this leads to the belief that the hydrophilicity/hydrophobicity of the compounds could be one of the major players influencing the stability of adsorption and affinity for leaching.

When HNEIs immobilized on membranes were incubated 3 h in plasma (Figure 3) versus HNEIs (“free”) in solution (Figure 1), the former better retained their capacity for HNE inhibition, since the HNEIs-PSU membranes presented higher HNE inhibition rates at 3 h than the HNEIs in solution. Therefore, it appears that the rapid clearance of HNEIs when free in plasma does not occur to the same extent when they are immobilized on the PSU membranes, as can be observed in Table 3. We surmise that HNEIs grafting into PSU membranes affects their affinity and/or accessibility to native plasma components, granting them a certain degree of protection and/or stability regarding the expected high rate of β-ring hydrolysis and off-target reactions. This bodes in favor of the potential endurance and efficacy of these HNEI-PSU membranes during a full HD session (usually between 180 and 240 min). Nevertheless, in the future, the implementation of this innovative therapeutic approach for CKD management must adhere to a finely tuned balance between the biomaterials’ bioactivity and the level of favorable HNE inhibition in vivo.

## 5. Conclusions

The synthesized HNEIs showed potent inhibitory capacity and selectivity for elastase. D4L-1 presented with the lowest HNE IC50 value, followed by D4L-2, and lastly by Sivelestat. The inhibitors showed biocompatibility in addition to high bioactivity. Their incorporation in PSU matrices (with added PVP) provided materials with inhibitory activity that were was also hemocompatible. The relative bioactivity of the compounds, when immobilized into PSU membranes, was as follows: D4L-2-PSU > Sivelestat-PSU > D4L-1-PSU. The degree of adsorption was related to the hydrophilic or lipophilic characteristics of the HNEIs and to their molecular size (D4L-1 performs better in a more lipophilic medium, while D4L-2 is the best inhibitor in a more hydrophilic milieu). The 4-oxo-β-lactam-based inhibitors showed to be more potent and more stable than the commercial inhibitor (Sivelestat). Nevertheless, for all HNEIs, the incorporation into the membrane favors their stability over time, showing great potential for application in HD membranes. Beyond the efficiency in the removal of uremic toxins and extra water, and in retaining essential metabolites, these bioactive PSU membranes have a specific biological activity that is inhibiting/reducing the inflammatory response, a dialysis-associated complication. This might prevent cardiovascular complications associated with HD, that are the major cause of death in ESRD patients on HD, thus improving their longevity and quality of life.

## Figures and Tables

**Table 1:** Chemical characteristics of D4L-1, D4L-2, and Sivelestat.

	D4L-1	D4L-2	Sivelestat
Molecular structure	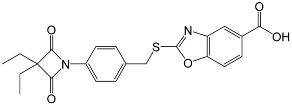	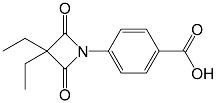	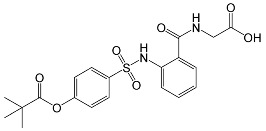
Molecular formula	C_22_H_20_N_2_O_5_S	C_14_H_15_NO_4_	C_20_H_22_N_2_O_7_S
Molecular weight (g mol^−1^)	424.47	261.28	434.46
tPSA (Å^2^)	96.27	74.68	138.87
CLogP	4.738	2.445	2.771
LogS	−5.157	−2.909	−4.846
pKa	3.704	3.714	3.783

tPSA: topological poral surface area; CLogP: calculated partition coefficient; LogS: aqueous solubility; pKa: acid dissociation constant as determined using ChemDraw Professional v. 18.2 (PerkinElmer Informatics, Waltham, MA, USA).

**Table 2:** Adsorption data for D4L-1, D4L-2, and Sivelestat-PSU doped membranes.

Adsorption Yield (%)		D4L-1	D4L-2	Sivelestat
[HNEI solution] nM	5	99.2	98.2	89.6
	25	99.0	95.3	90.3
	50	99.1	96.3	92.9
	250	99.2	96.4	92.5
	500	99.6	92.0	93.5
	1000	99.4	86.5	89.8
Adsorption Capacity (K_F_) mg g^−1^		98.5	0.824	3.56
Intensity of Adsorption (n)		0.89	1.36	0.96

HNEI: human neutrophil elastase inhibitor; adsorption yield (%) = [HNEI mass in initial solution (ng)—[HNEI mass in final solution (ng)]/HNEI mass in initial solution (ng) × 100; K_F_ and n, determined from the application of the Freundlich adsorption isotherm linearized equation: ln(Q_e_) = ln(K_F_) + 1/n ln(C_e_), see Figure S5 of Supplementary Materials.

**Table 3:** Bioactivity comparison of HNEIs in solution versus immobilized on PSU membranes after 3 h incubation in diluted plasma or in HEPES buffer.

% of HNE Inhibition Variation after 3 h Incubation In:	HNEIs in Solution			HNEIs Immobilized in PSU		
	D4L-1	D4L-2	Sivelestat	D4L-1	D4L-2	Sivelestat
Diluted plasma (50%; PBS, pH 7.4)	−86.6 ± 1.6	−26.8 ± 2.9	−27.0 ± 4.1	−12.0 ± 1.8	−2.0 ± 0.7	−19.5 ± 2.5
HEPES 0.1M buffer	0.3 ± 0.3	−2.0 ± 0.4	0.3 ± 2.2	0.0 ± 0.7	−7.5 ± 2.5	−1.3 ± 7.8

Data obtained from at least 3 independent experiments (performed in triplicate) are presented as mean ± SEM (standard error of the mean). % of HNE inhibition variation refers to the difference between 0 and 3 h incubation time for the 100× IC50 HNEIs solution and for the HNEI-PSU 1000 nM membranes, respectively. HNE, human neutrophil elastase; HNEIs, human neutrophil elastase inhibitors; PSU, polysulfone.

## Data Availability

The data presented in this study are available on request from the corresponding author.

## Supplementary Materials

The following supporting information can be downloaded at: https://www.mdpi.com/article/10.3390/membranes13010089/s1, several elements (3 Tables and 8 Figures) are provided as supporting information. Table S1: Details of the MRM conditions applied in the UHPLC-MS/MS analysis. Figure S1: Nonlinear regression model for D4L-1, D4L-2, and Sivelestat human neutrophil elastase (HNE) inhibition capacity, assessed by the fluorometric assay to measure HNE activity, and the half maximal inhibitory concentration estimated with a 95% confidence interval [IC50_HNE_ (CI95%), embedded table]. Dotted lines represent the 95% confidence band for data obtained from at least 5 independent experiments performed in triplicate. HNE activity was standardized considering that HNEIs’ vehicle (2.5% DMSO) samples corresponded to maximum enzyme activity (100.0%) for each assay. Figure S2: Hemocompatibility tests for D4L-1, D4L-2, and Sivelestat solutions, assessed by plasma’s hemoglobin (Hb) concentration (A) and platelet (PLT) activation (B). Data obtained from 3 independent experiments (performed in duplicate) are presented as mean ± SEM (standard error of the mean). Dashed line represents the cut-off value for hemolytic behavior as recommended by the International Standard ISO 10993-4 (2017). Brackets denote the statistical analysis results between HNEI concentrations groups’ comparison within each HNEI, and the embedded table the comparison between D4L-1, D4L-2, and Sivelestat groups within each concentration (one-way ANOVA test for A and Kruskal–Wallis test for B); *p* < 0.05 was considered statistically significant. For each assay, Hb_plasma_ and PLT_activation_ were standardized establishing that HNEIs’ vehicle (0.125% DMSO) samples corresponded to zero (none). HNEI, human neutrophil elastase inhibitor; IC50, half maximal inhibitory concentration. Figure S3: Representative optical microscopy images of Wright-stained blood smears after 3 h incubation at 37 °C of blank (blood only), HNEIs’ vehicle (0.125% DMSO), and HNEIs in different concentrations (100× magnification). HNEI, human neutrophil elastase inhibitor; IC50, half maximal inhibitory concentration. Figure S4: Human neutrophil elastase (HNE) inhibition of D4L-1, D4L-2, and Sivelestat when incubated with HEPES 0.1M buffer, pH 7.4, assessed by the HNE activity assay after incubations of 0, 15, 30, 60, and 180 min at 37 °C. Data obtained from at least 3 independent experiments (performed in triplicate) are presented as mean ± SEM (standard error of the mean). Dotted line represents the IC50 value of the respective HNEI. Brackets denote the statistical analysis results of time of incubation comparison for the respective solution concentration of each HNEI (one-way ANOVA test); the embedded table shows, for each time point, the comparison between D4L-1, D4L-2, and Sivelestat groups within each concentration, on the left side; the comparison the between concentration groups within each HNEI, in the middle (one-way ANOVA test or Student’s *t* test); and the effect of the interaction between concentration and type of HNEI, on the right (two-way ANOVA test); *p* < 0.05 was considered statistically significant. For each assay, HNE inhibition was standardized establishing that HNEIs’ vehicle (0.125% DMSO) samples corresponded to zero (minimum). HNEI, human neutrophil elastase inhibitor; IC50, half maximal inhibitory concentration. Table S2: Complement to Figure 1: For each time point, the comparison between D4L-1, D4L-2, and Sivelestat groups within each concentration, on the left side; the comparison between the concentration groups within each HNEI (one-way ANOVA test), in the middle; and the effect of the interaction between concentration and type of HNEI (two-way ANOVA test), on the right, according to HNEIs’ incubations with plasma (top) or diluted plasma (bottom)**.** Figure S5: Linearization of Freundlich adsorption isotherms for HNEI-PSU doped membranes [Ln(q_e_) = Ln(K_F_) + 1/n Ln(C_e_)]. Data obtained from 3 independent experiments are presented as mean; dotted lines represent the 95% confidence band for each linear regression fit line. HNEI, human neutrophil elastase inhibitor; PSU, polysulfone; q_e_, mass of adsorbate adsorbed (mg) per mass of adsorbent (g) at equilibrium; K_f_, Freundlich constant; Ce, aqueous-phase adsorbate concentration at equilibrium (mg L^−1^). Figure S6: Hemocompatibility tests for HNEI-PSU membranes, assessed by plasma’s hemoglobin (Hb) concentration (A) and platelet (PLT) activation (B). Data obtained from 3 independent experiments (performed in duplicate) are presented as mean ± SEM (standard error of the mean). Dashed line represents the cut-off value for hemolytic behavior as recommended by the International Standard ISO 10993-4 (2017). Brackets denote the statistical analysis results between HNEI concentrations groups’ comparison within each HNEI, and the embedded table the comparison between D4L-1, D4L-2, and Sivelestat groups within each concentration (one-way ANOVA test for A and Kruskal–Wallis test for B); *p* < 0.05 was considered statistically significant. For each assay, Hb_plasma_ and PLT_activation_ were standardized establishing the HNEI-PSU vehicle (2.5% DMSO) samples corresponded to zero (none). HNEI, human neutrophil elastase inhibitor; PSU, polysulfone. Figure S7: Representative optical microscopy images of Wright-stained blood smears after 3 h incubation at 37 °C with blood of blank-PSU, vehicle-PSU (2.5% DMSO), and HNEIs-PSU membranes corresponding to different HNEIs’ initial solutions concentrations (100× magnification). HNEI, human neutrophil elastase inhibitor; PSU, polysulfone. Figure S8: Human neutrophil elastase (HNE) inhibition of plasma (diluted 50%) and HEPES 0.1M buffer, pH 7.4 supernatants, assessed by the HNE activity assay after 180 min incubation at 37 °C with HNEI-PSU membranes. Data obtained from at least 3 independent experiments (performed in triplicate) are presented as mean ± SEM (standard error of the mean). Dotted line represents the IC50 value of the respective HNEI in HEPES 0.1M, pH 7.4 buffer. Brackets denote the statistical analysis results of comparison of HNEI-PSU membranes incubated with respective initial solution concentrations of each HNEI (one-way ANOVA test), ** *p* < 0.01, *** *p* < 0.001 between that [HNEI solution] and all the preceding concentrations; ^££^ *p* < 0.01 between that [HNEI solution] and 5/25 nM; ^§^ *p* < 0.05 between that HNEI solution and 5/25 nM; ^$^ *p* < 0.05, between that [HNEI solution] and 5 nM (Bonferroni post hoc test); and in the embedded table, the comparison between D4L-1, D4L-2, and Sivelestat groups within supernatants (one-way ANOVA test); *p* < 0.05 was considered statistically significant. For each assay, HNE inhibition was standardized establishing the HNEI-PSU vehicle (2.5% DMSO) samples corresponded to zero (minimum). HNEI, human neutrophil elastase inhibitor; PSU, polysulfone. Table S3: Complement to Figure 3: For each time point, the comparison between D4L-1-PSU, D4L-2-PSU, and Sivelestat-PSU membranes within each concentration, on the left side, the comparison between the concentration groups within each HNEI-PSU biomaterial (one-way ANOVA test); in the middle, the effect of the interaction between concentration of HNEI initial solution and type of HNEI-PSU membrane (two-way ANOVA test); on the right, according to HNEIs’ incubations with plasma (top) or diluted plasma (bottom).
