# Supplementary material for: Polysulfone Membranes Doped with Human Neutrophil Elastase Inhibitors: Assessment of Bioactivity and Biocompatibility
Source: Membranes (Basel). 2023 Jan 10;13(1):89. doi: 10.3390/membranes13010089 (PMC9861744; doi:10.3390/membranes13010089)
Supplement: Supplementary file 1 [file membranes-13-00089-s001.zip › Table S1.pdf]

Table S1: **Details of the MRM conditions applied in the UHPLC-MS/MS analysis.**

Compound	[M-H]-	MRM1	MRM2	Cone Voltage (V)	Collision Energy (eV)
**D4L-1**	423	150	246	40	20
**D4L-2**	260	216	188	30	20
**Sivelestat**	433	157	389	40	20
