# Supplementary material for: Polysulfone Membranes Doped with Human Neutrophil Elastase Inhibitors: Assessment of Bioactivity and Biocompatibility
Source: Membranes (Basel). 2023 Jan 10;13(1):89. doi: 10.3390/membranes13010089 (PMC9861744; doi:10.3390/membranes13010089)
Supplement: Supplementary file 1 [file membranes-13-00089-s001.zip › Table S2.pdf]

Table S2 – **Complement to Figure 1:** For each time point, the comparison between D4L-1, D4L-2, and Sivelestat groups within each concentration, on the left side; the comparison between the concentration groups within each HNEI (one-way ANOVA test), in the middle; and the effect of the interaction between concentration and type of HNEI (two-way ANOVA test), on the right, according to HNEIs' incubations with plasma (top) or diluted plasma (bottom).

Plasma	*D4L-1 vs. D4L-2 vs. Sivelestat, p*				*100x vs. 10x vs. 1x vs. 0.1x vs. 0.1x IC50s, p*		
Time (min.)	**100x IC50**	**10x IC50**	**1x IC50**	**0.1x IC50**	**D4L-1**	**D4L-2**	**Sivelestat**
0	<0.001	<0.001	0.153	0.347	<0.001	0.033	0.534
15	<0.001	<0.001	0.132	0.141	<0.001	<0.001	0.071
30	<0.001	0.001	0.338	0.639	<0.001	0.012	0.375
60	<0.001	0.078	0.144	0.641	<0.001	0.040	0.002
180	0.572	0.088	0.135	0.611	0.064	0.026	0.001

Diluted Plasma	***D4L-1 vs. D4L-2 vs. Sivelestat, p***				***100x vs. 10x vs. 1x vs. 0.1x vs. 0.01x IC50, p***		
Time (min.)	**100x IC50**	**10x IC50**	**1x IC50**	**0.1x IC50**	**D4L-1**	**D4L-2**	**Sivelestat**
0	<0.001	<0.001	0.041	0.964	<0.001	<0.001	0.808
15	<0.001	<0.001	0.030	0.548	<0.001	<0.001	0.190
30	<0.001	<0.001	0.028	0.309	<0.001	<0.001	0.002
60	<0.001	0.001	0.040	0.146	<0.001	<0.001	0.017
180	0.037	0.645	0.630	0.954	<0.001	0.001	0.276

IC50, half maximal inhibitory concentration. p<0.05 was considered statistically significant (ANOVA one-way test).
