# Supplementary material for: Polysulfone Membranes Doped with Human Neutrophil Elastase Inhibitors: Assessment of Bioactivity and Biocompatibility
Source: Membranes (Basel). 2023 Jan 10;13(1):89. doi: 10.3390/membranes13010089 (PMC9861744; doi:10.3390/membranes13010089)
Supplement: Supplementary file 1 [file membranes-13-00089-s001.zip › Table S3.pdf]

Table S3 – **Complement to Figure 3:** For each time point, the comparison between D4L-1-PSU, D4L-2-PSU, and Sivelestat-PSU mem-branes within each concentration, on the left side, the comparison between the concentration groups within each HNEI-PSU biomaterial (one-way ANOVA test); in the middle, the effect of the interaction between concentration of HNEI initial solution and type of HNEI-PSU membrane (two-way ANOVA test); on the right, according to HNEIs' incubations with plasma (top) or diluted plasma (bottom).

Plasma	*D4L-1-PSU vs. D4L-2PSU vs. Sivelestat-PSU, p*						*5 vs. 25 vs. 50 vs. 250x vs. 500 vs. 1000, p*		
Time (min.)	**5**	**25**	**50**	**250**	**500**	**1000**	**D4L-1-PSU**	**D4L-2-PSU**	**Sivelestat-PSU**
0	0.446	0.148	0.224	<0.001	<0.001	<0.001	<0.001	0.084	0.533
180	0.020	0103	<0.001	0.010	<0.001	<0.001	<0.001	0.014	0.763

HEPES	***D4L-1 vs. D4L-2 vs. Sivelestat, p***						***100x vs. 10x vs. 1x vs. 0.1x vs. 0.01x IC50, p***		
Time (min.)	**5**	**25**	**50**	**250**	**500**	**1000**	**D4L-1**	**D4L-2**	**Sivelestat**
0	0.218	0.344	0.147	0.021	0.005	<0.001	0.161	<0.001	0.054
180	0.023	0.125	0.028	<0.001	<0.001	0.507	0.002	0.042	<0.001

PSU, polysulfone. p<0.05 was considered statistically significant (ANOVA one-way test).
