# Supplementary figures and images for: Polysulfone Membranes Doped with Human Neutrophil Elastase Inhibitors: Assessment of Bioactivity and Biocompatibility
Source: Membranes (Basel). 2023 Jan 10;13(1):89. doi: 10.3390/membranes13010089 (PMC9861744; doi:10.3390/membranes13010089)

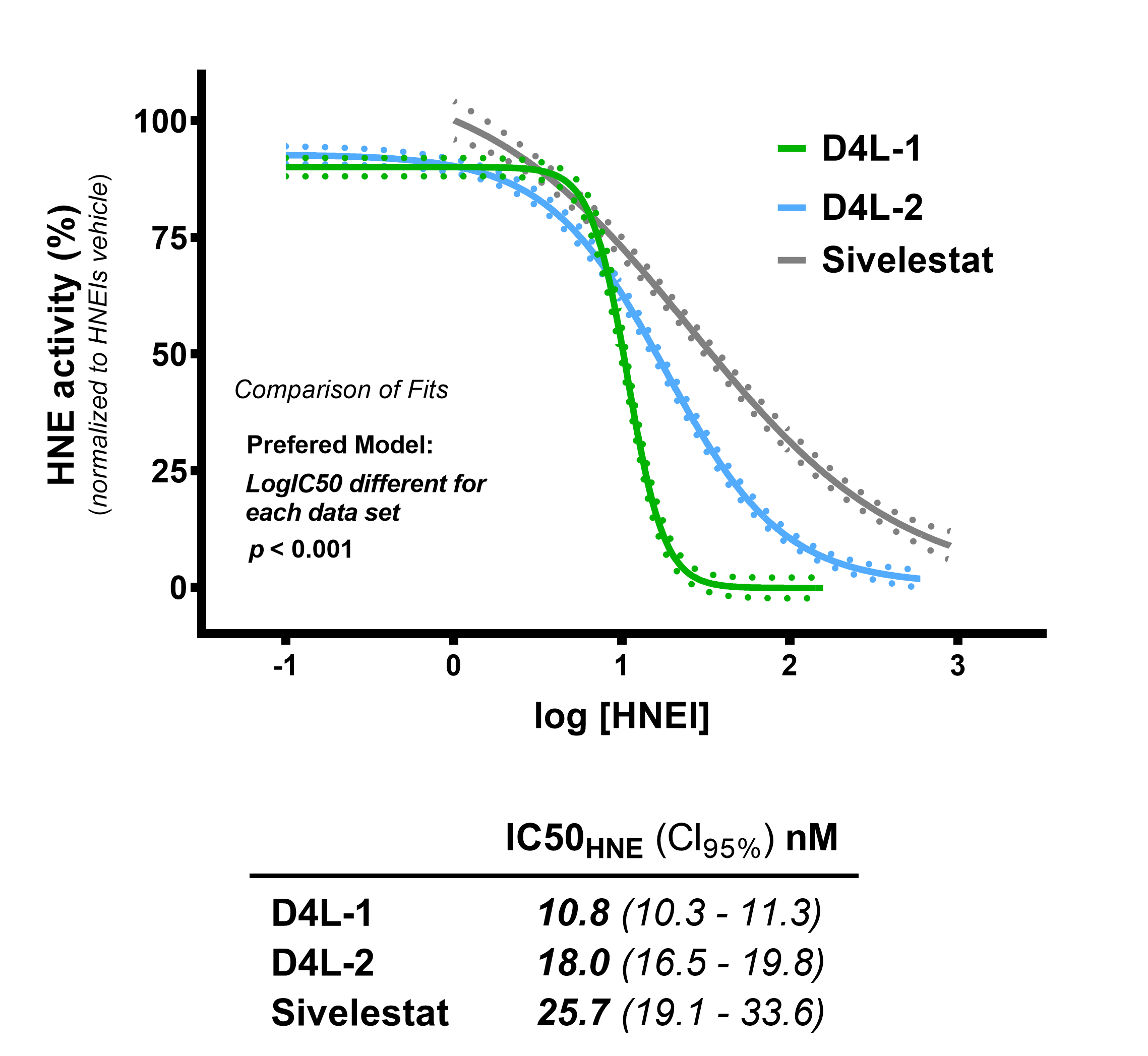

Supplement: Supplementary file 1 [file membranes-13-00089-s001.zip › Figure S1.tif]

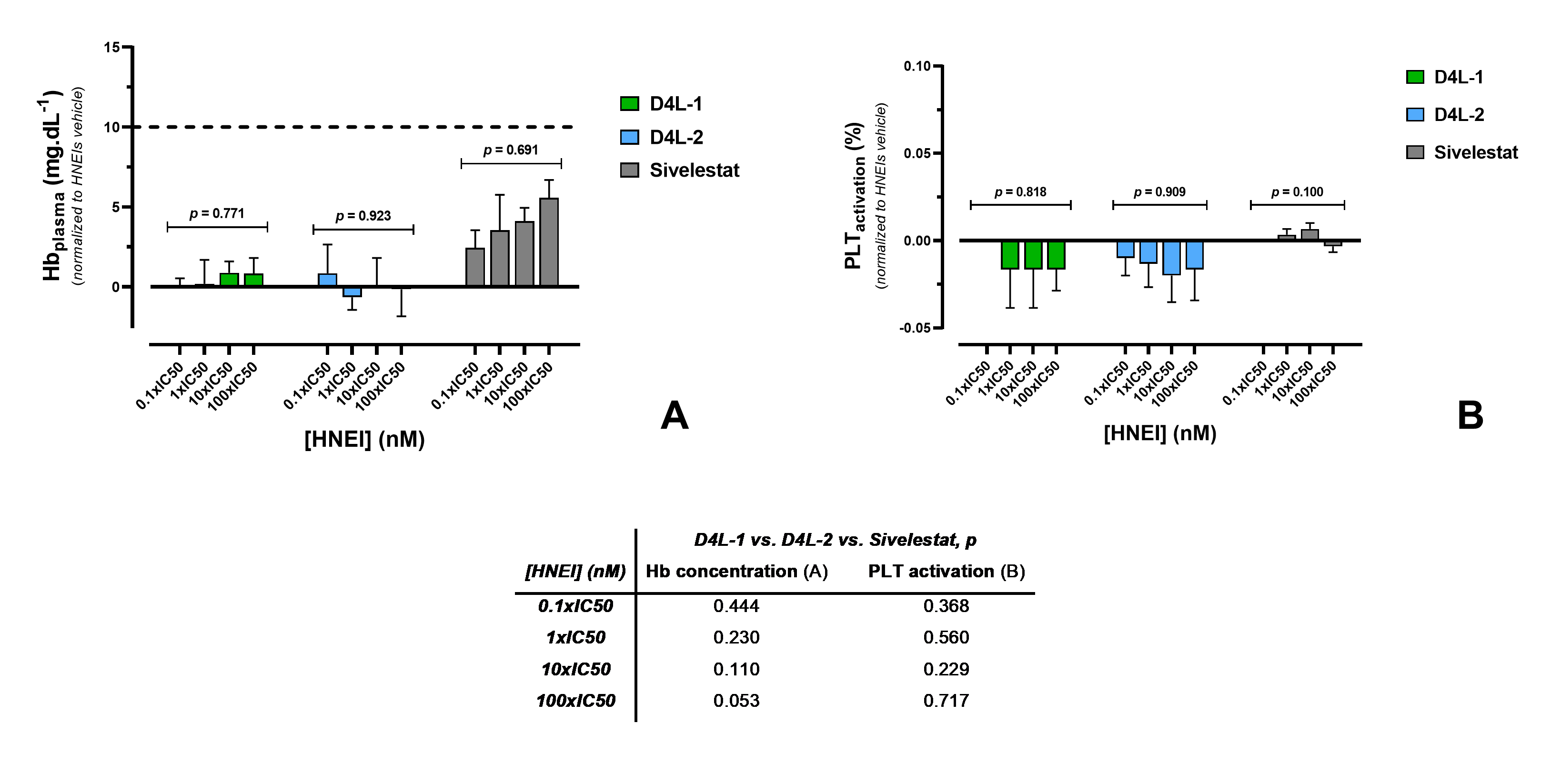

Supplement: Supplementary file 1 [file membranes-13-00089-s001.zip › Figure S2.tif]

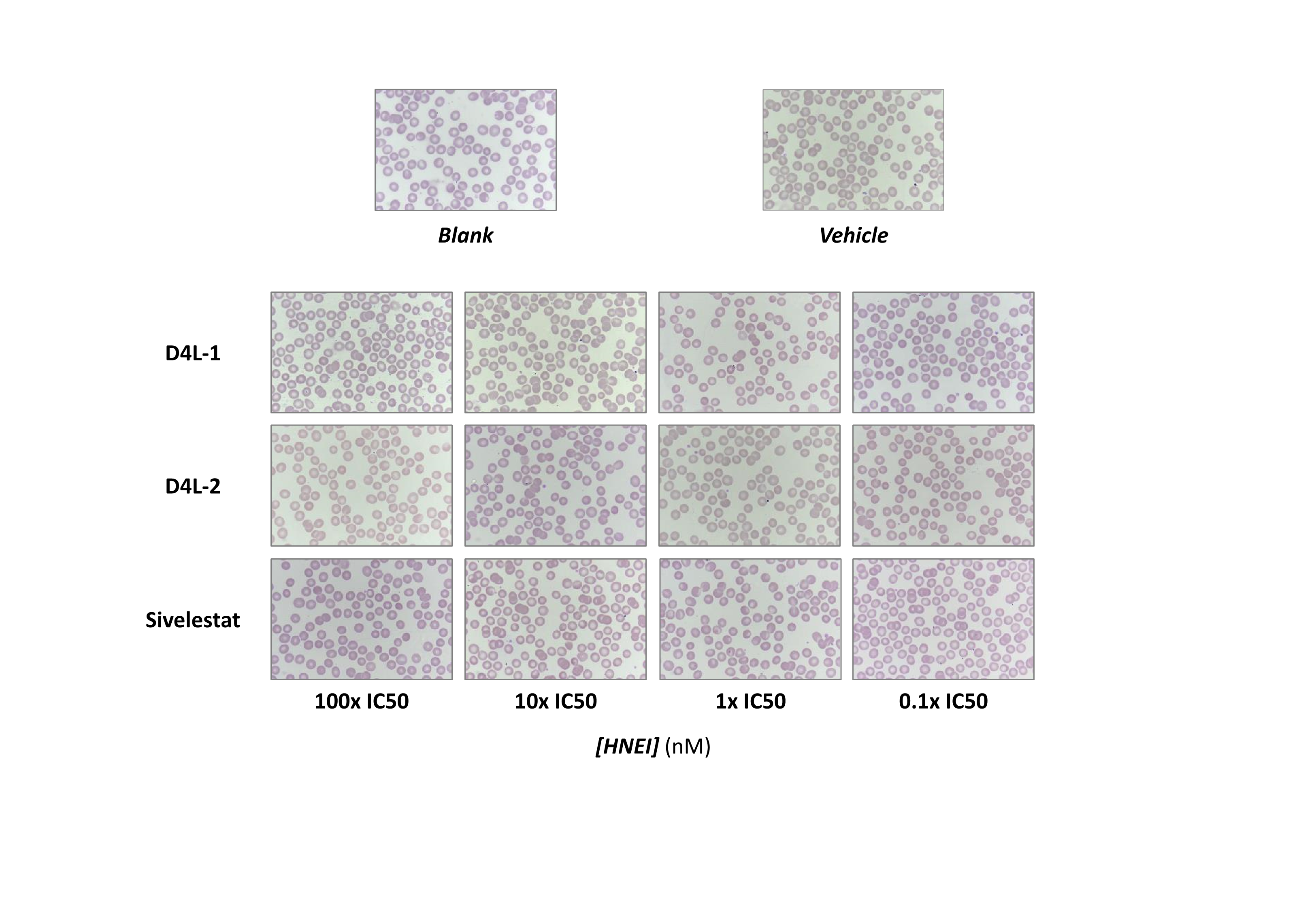

Supplement: Supplementary file 1 [file membranes-13-00089-s001.zip › Figure S3.tif]

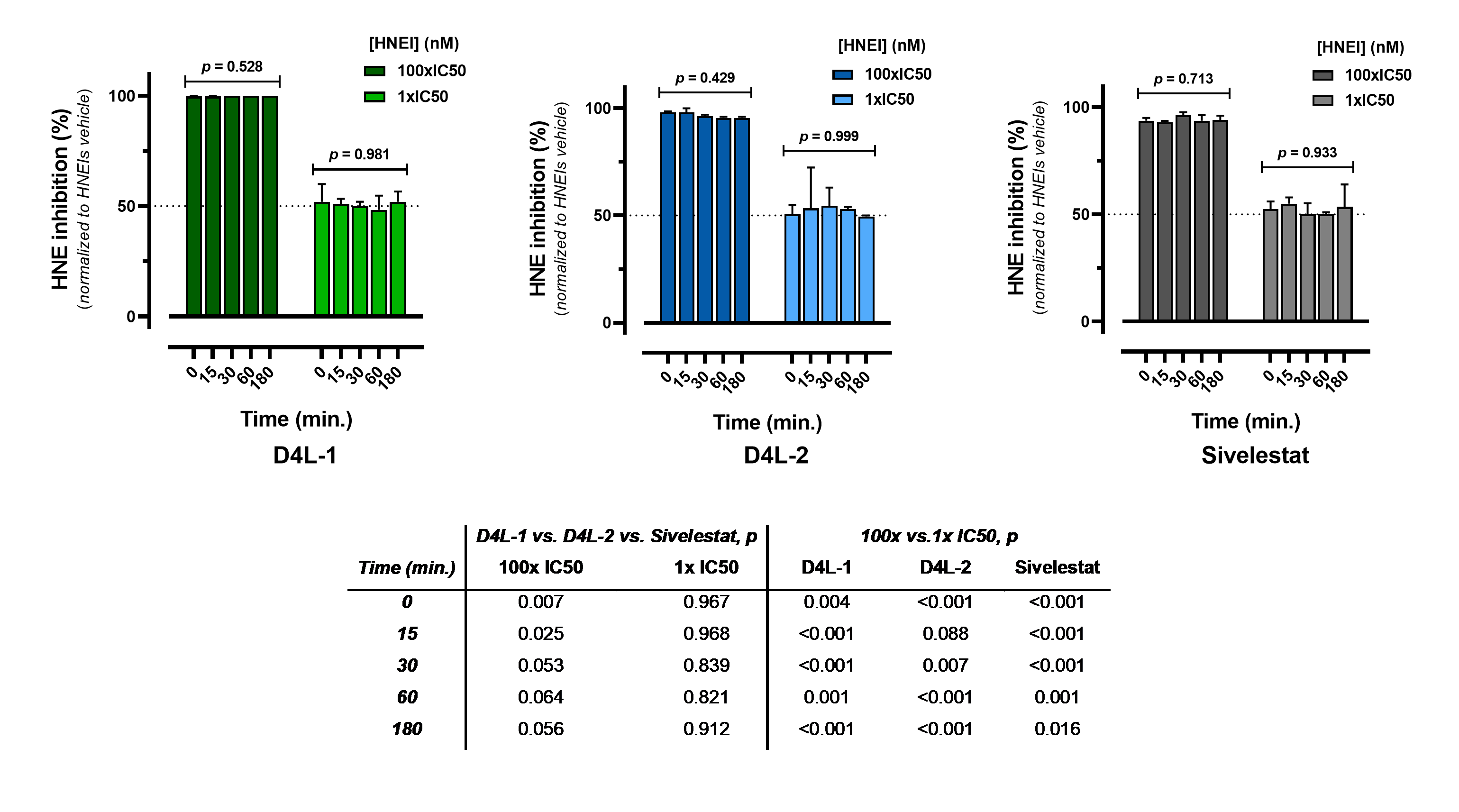

Supplement: Supplementary file 1 [file membranes-13-00089-s001.zip › Figure S4.tif]

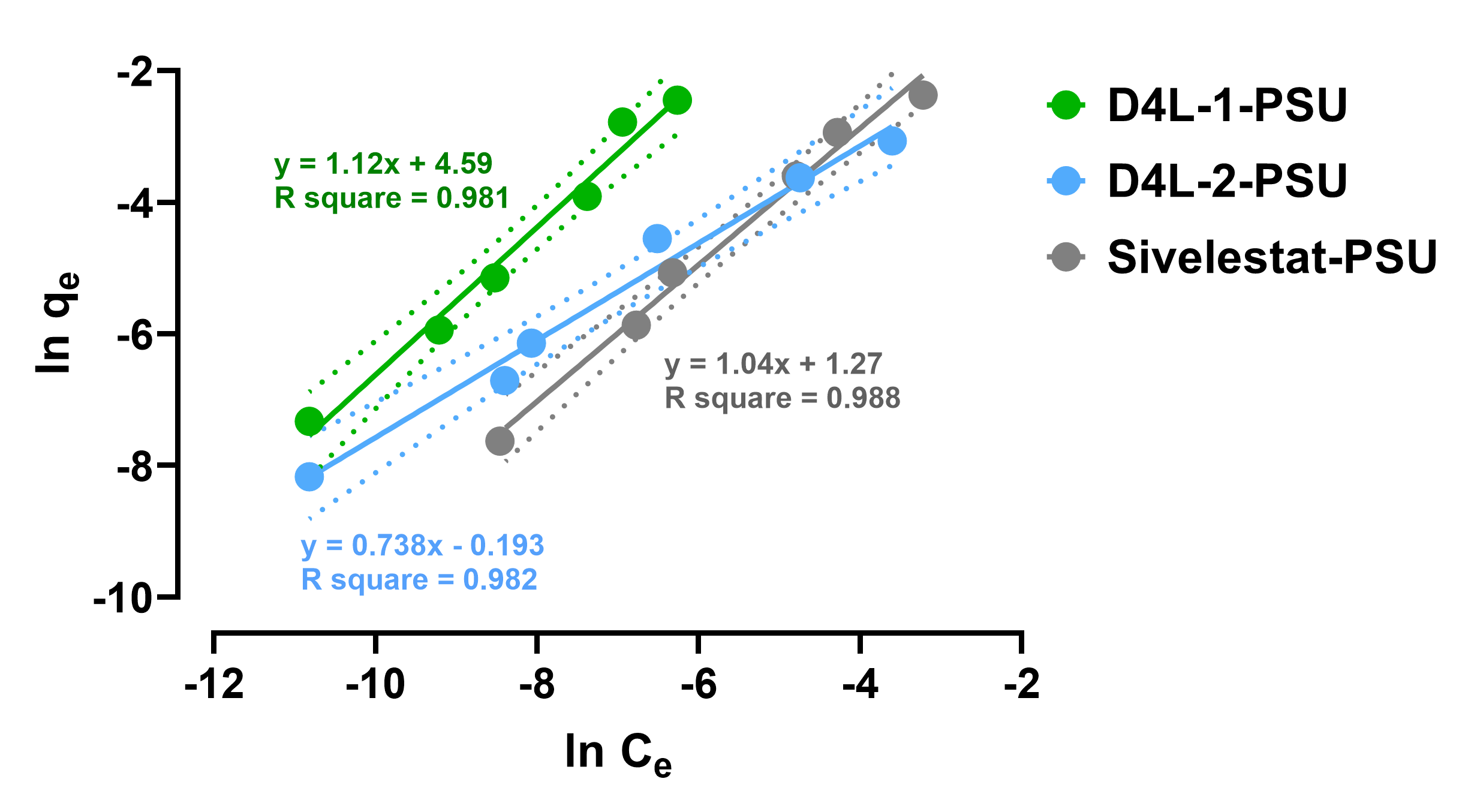

Supplement: Supplementary file 1 [file membranes-13-00089-s001.zip › Figure S5.tif]

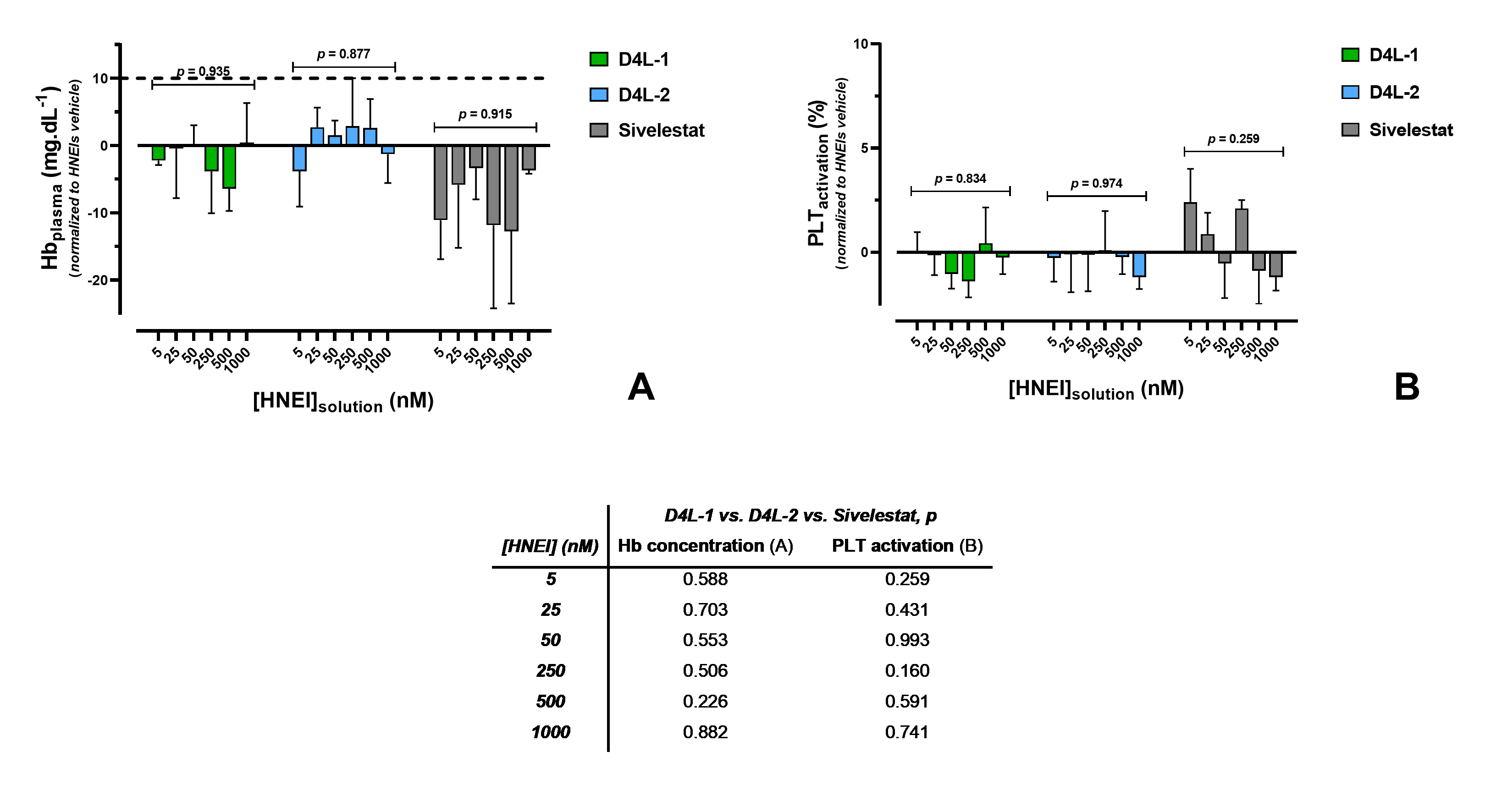

Supplement: Supplementary file 1 [file membranes-13-00089-s001.zip › Figure S6.tif]

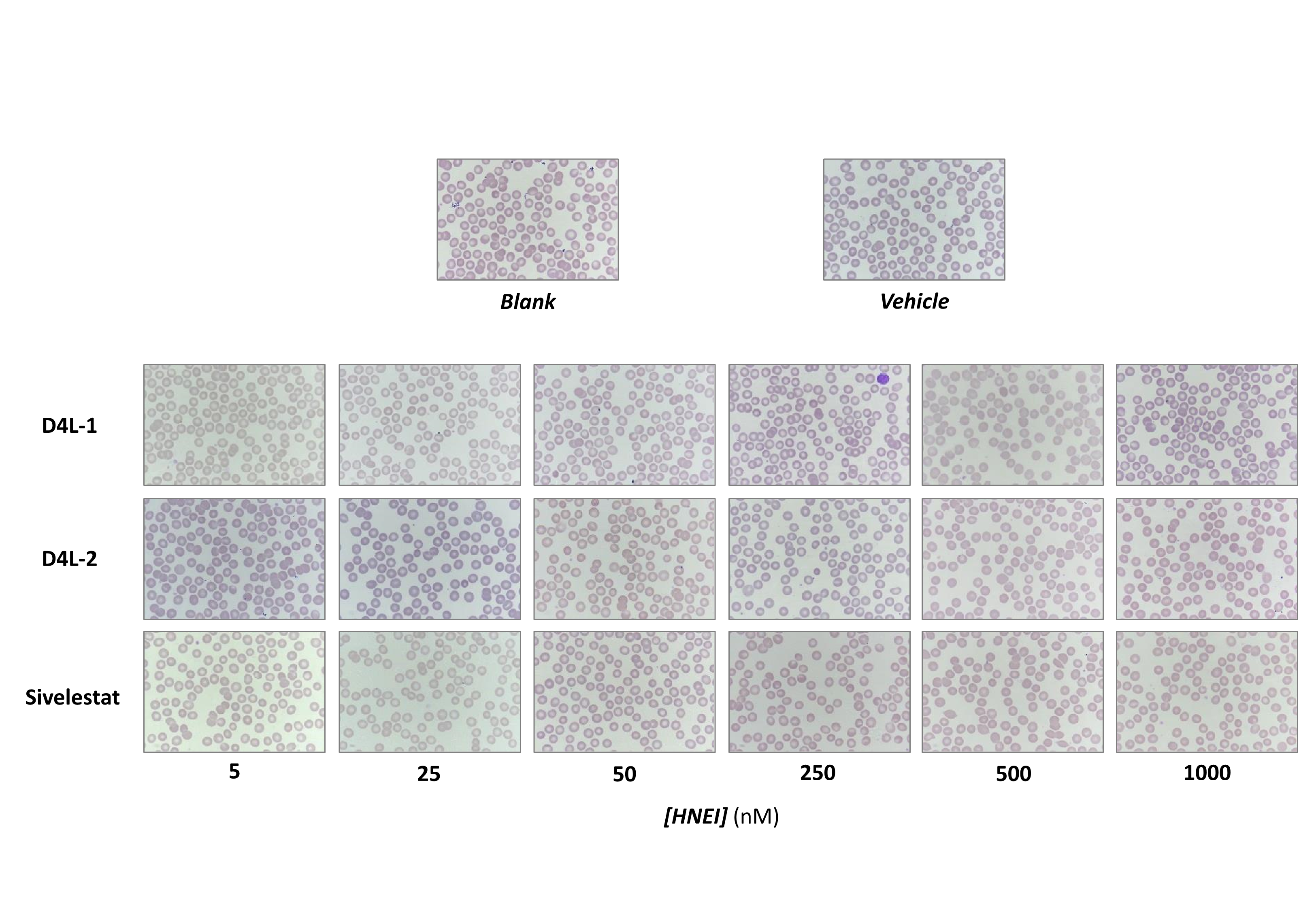

Supplement: Supplementary file 1 [file membranes-13-00089-s001.zip › Figure S7.tif]

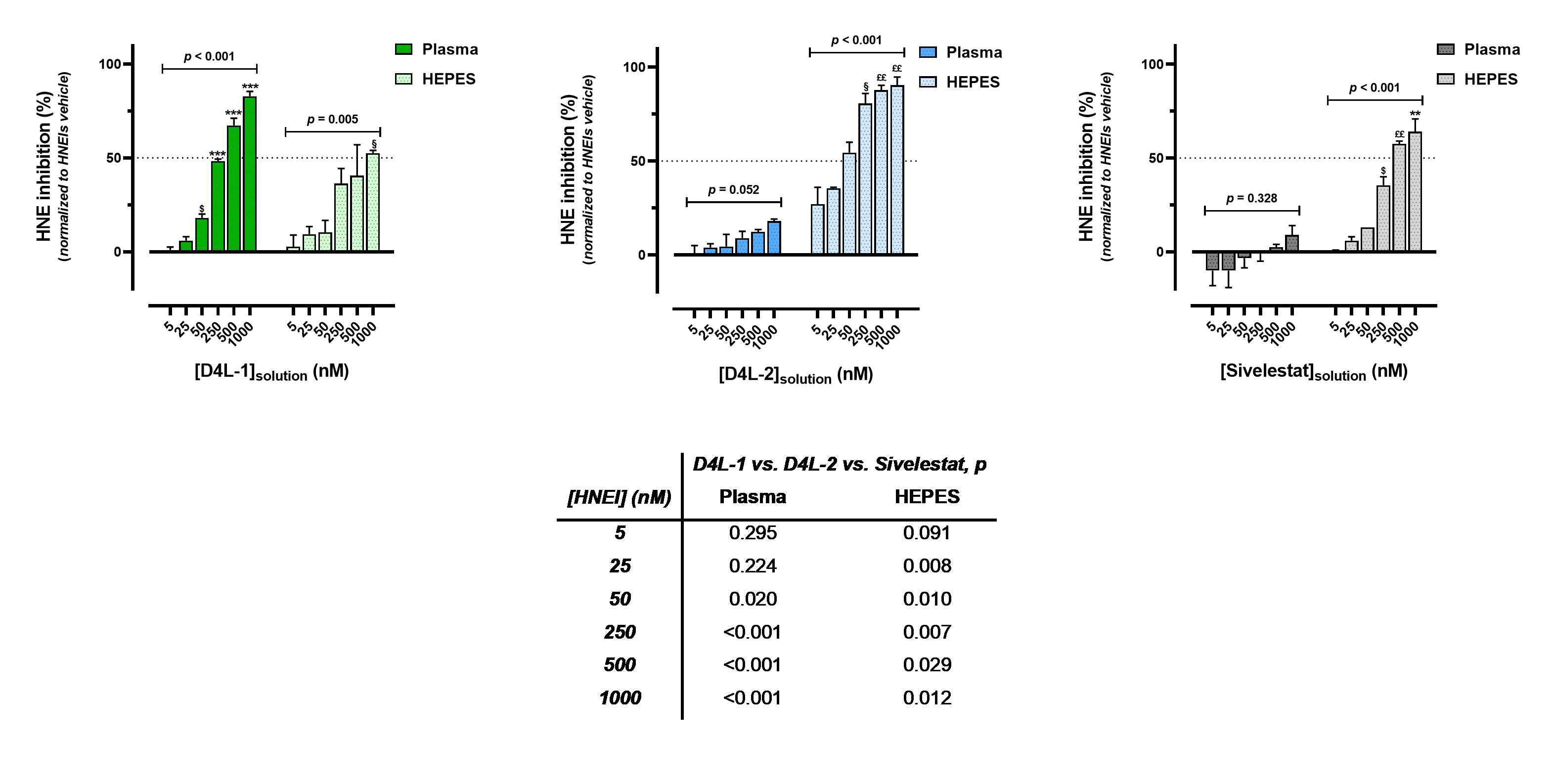

Supplement: Supplementary file 1 [file membranes-13-00089-s001.zip › Figure S8.tif]
